# Targeted Disruption in Mice of a Neural Stem Cell-Maintaining, KRAB-Zn Finger-Encoding Gene That Has Rapidly Evolved in the Human Lineage

**DOI:** 10.1371/journal.pone.0047481

**Published:** 2012-10-10

**Authors:** Huan-Chieh Chien, Hurng-Yi Wang, Yi-Ning Su, Kuan-Yu Lai, Li-Chen Lu, Pau-Chung Chen, Shih-Feng Tsai, Chung-I Wu, Wu-Shiun Hsieh, Che-Kun James Shen

**Affiliations:** 1 Department of Life Sciences and Institute of Genome Sciences, National Yang-Ming University, Taipei, Taiwan, Republic of China; 2 Institute of Molecular Biology, Academia Sinica, Taipei, Taiwan, Republic of China; 3 Institute of Clinical Medicine, National Taiwan University College of Medicine, Taipei, Taiwan, Republic of China; 4 Department of Medical Genetics, National Taiwan University Hospital, Taipei, Taiwan, Republic of China; 5 Institute of Occupational Medicine and Industrial Hygiene, National Taiwan University College of Public Health, Taipei, Taiwan, Republic of China; 6 Division of Molecular and Genomic Medicine, National Health Research Institutes, Zhunan, Miaoli County, Taiwan, Republic of China; 7 Department of Ecology and Evolution, University of Chicago, Chicago, Illinois, United States of America; 8 Department of Pediatrics, National Taiwan University Hospital and National Taiwan University College of Medicine, Taipei, Taiwan, Republic of China; University of South Florida, United States of America

## Abstract

Understanding the genetic basis of the physical and behavioral traits that separate humans from other primates is a challenging but intriguing topic. The adaptive functions of the expansion and/or reduction in human brain size have long been explored. From a brain transcriptome project we have identified a KRAB-Zn finger protein-encoding gene (M003-A06) that has rapidly evolved since the human-chimpanzee separation. Quantitative RT-PCR analysis of different human tissues indicates that M003-A06 expression is enriched in the human fetal brain in addition to the fetal heart. Furthermore, analysis with use of immunofluorescence staining, neurosphere culturing and Western blotting indicates that the mouse ortholog of M003-A06, Zfp568, is expressed mainly in the embryonic stem (ES) cells and fetal as well as adult neural stem cells (NSCs). Conditional gene knockout experiments in mice demonstrates that Zfp568 is both an NSC maintaining- and a brain size-regulating gene. Significantly, molecular genetic analyses show that human M003-A06 consists of 2 equilibrated allelic types, H and C, one of which (H) is human-specific. Combined contemporary genotyping and database mining have revealed interesting genetic associations between the different genotypes of M003-A06 and the human head sizes. We propose that M003-A06 is likely one of the genes contributing to the uniqueness of the human brain in comparison to other higher primates.

## Introduction

In less than the 3 million years since our divergence from chimpanzee, the human brain has roughly tripled in volume, a fascinating fact that cannot be explained simply by the increase of the human body size [1]. From a genetic point of view, the relatively larger and more complex human brain most likely arose from human-specific functions of certain genes underlying the biology of brain development [2], [3]. The rapid evolution in the expression levels of genes in the human brain has been suggested to be partly responsible for the phenotypic differences between human and apes [4], [5], [6], [7]. However, although genetic factors in modern humans are known to induce variations in brain phenotypes such as size, organization, cognitive abilities, personality traits, and perhaps even psychiatric conditions [2], [8], [9], little is known about the genetic changes occurring in the human lineage that are responsible for its markedly altered brain phenotypes, e.g. the pronounced brain expansion, in comparison to other higher primates.

Research on the genetic mechanisms governing the variation in brain volumes of the human population may contribute to a better understanding of the evolution of the human brain and cognition in comparison to other higher primates. The search for the genetic basis of human brain evolution has relied mainly on studies of human brain malformations. For example, several studies have identified at least 7 different genes of which mutations lead to autosomal recessive primary microcephaly (MCPH), a class of rare disorders of human brain development [10], [11]. Interestingly, the brain sizes of the affected MCPH individuals are smaller and similar to those of the early hominids, suggesting that MCPH genes might play a role in the evolutionary expansion of the primate brains. Nevertheless, there is no known genetic correlation between the MCPH genes and the relatively large head/brain size of humans in comparison to the other primates [12]. Surprisingly, although humans have larger brains than other primates, recent studies of human fossils have also shown that the average volume of the human brain has decreased from 1,500 to 1,350 cubic centimeters over the last 250,000 years [2], [13]. It is still debatable as to why modern human brains are shrinking, and both genetic and environmental changes may have contributed to the startling decline of our brain size.

Previously, we carried out a systematic brain transcriptome comparison among the human and other primates including the chimpanzee and an Old World Monkey, the macaque [14]. Unexpectedly, we found that genes expressed in the primate brains have, as a whole, evolved significantly more slowly at their nonsynonymous sites than non-brain genes. In humans, the average rate of protein change for brain-expressed genes is only 62.9% of the genome average. We attribute this to the more complex molecular interaction network in the human brain [14]. In interesting contrast, rapid evolution in the expression levels of genes expressed in the human brain has been observed [5]. Also, quite a few nervous system genes do display significantly higher rates of protein evolution in primates than in rodents. This acceleration of protein evolution is most prominent in the lineage leading from the ancestral primates to humans [15]. Interestingly, several additional fast-evolving brain genes have also been identified through our transcriptome analysis in combination with our later sequencing and bioinformatic analysis of specific genomic regions and cDNAs from the orangutan, gibbon, and baboon (H.-C. Chien, unpublished). Significantly, one of these brain genes appeared to have episodically evolved in the human lineage since its separation from the chimpanzee.

We report below the characteristics of this gene which encodes a KRAB-Zn finger protein in embryonic stem cells, neural stem cells, and in the early fetal brain. We demonstrate, by a gene-targeting approach in mice, that this gene is functionally involved in the maintenance/self-renewal of NSCs and regulation of the fetal brain size. These molecular and cellular studies together with the correlation between different genotypes of this gene and the head sizes within a specific population as well as among different human populations suggest that this gene may play a unique role in human-specific brain development.

## Results

### Identification of a Gene Expressed in the Brain that has Rapidly Evolved in the Human Lineage

To identify genes expressed in the brain that have been fast-evolving in the human lineage after separation from our closest relative, the chimpanzee, we used codeml implemented in Phylogenetic Analysis by Maximum Likelihood (PAML) software [16] to estimate the numbers of synonymous and nonsynonymous changes and to measure the rates (Ks and Ka, see Materials and Methods) of coding sequence evolution in the human and chimpanzee lineages using the Old World Monkey (OWM) as an outgroup. In general, if Ka is significantly greater than Ks, positive selection of the gene(s) is suggested. However, since evolution of the genes expressed in the brain is highly constrained [14], it may not be realistic to search for brain genes with Ka > Ks. We therefore ranked genes with an excess of nonsynonymous changes in the human lineage.

**Figure 1:**
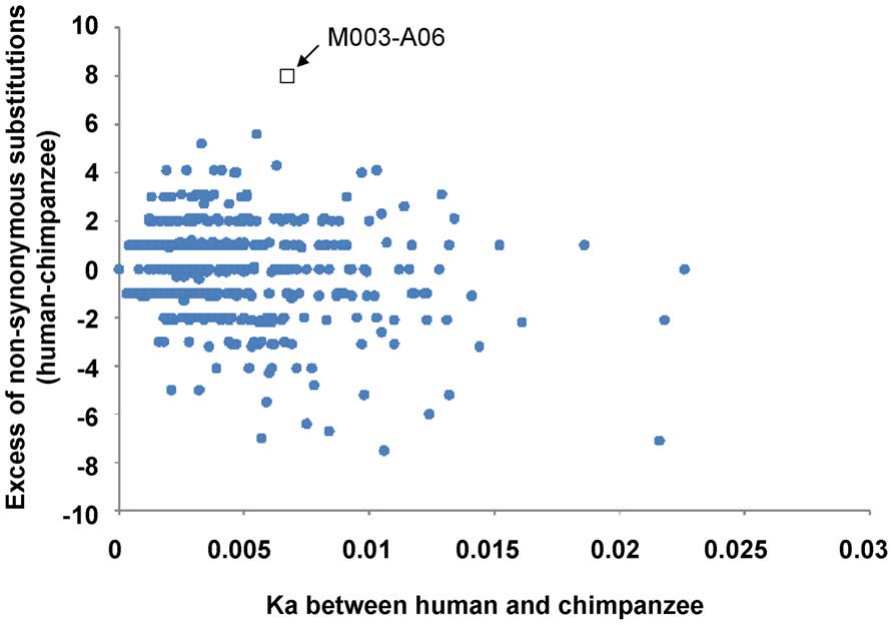
Plot of the excess of nonsynonymous substitutions between human and chimpanzee against Ka. The Ka values (X-axis) and the numbers of the excess nonsynonymous substitutions (Y-axis) between human and chimpanzee for 1,668 brain expressed genes were estimated by the maximum likelihood method implemented in PAML and plotted. The excess was calculated as [(number of changes in human) - (number of changes in chimpanzee)]. Thus, a positive value indicates more changes in the human lineage than in the chimpanzee lineage and a negative value means more changes in the chimpanzee lineage. The arrow points to the gene M003-A06, which has the highest number of excess nonsynonymous substitutions in the human lineage.

When the Ka values were plotted against the differences in the numbers of nonsynonymous changes between human and chimpanzee, one gene (M003-A06) stood out with the greatest number of nonsynonymous substitutions, 8, in excess in the human lineage among the 1,668 genes surveyed (**Figure 1**). The M003-A06 homologue in mouse encoded a KRAB-zinc finger protein Zfp568, which was shown to be important for early mouse embryo development [17]. It should be noted here that initially we could not find a complete gene annotation of M003-A06 in either NCBI or UCSC database. However, a human ortholog of the mouse Zfp568, namely ZNF568, showed up later. One of the six ZNF568 variants (variant 5, NM_001204838.1) was identical to the M003-A06 H-type that we have identified (see below). However, the other variants in the databases exhibited different exon/intron numbers or distinct C-terminal sequences. For example, 3.3 Kb of the C-terminal region of variant 1 only matched to a continuous stretch of genomic DNA but not to any EST. Thus, the other Zfp568 variants might be derived from alternative splicing and/or due to flaws during the automatic annotation process. In any case, the human M003-A06 gene contained 10 exons and was located on chromosome 19 at 19q13.1∼19q13.2, a region containing many KRAB-zinc finger protein-encoding genes. To determine whether M003-A06 was the only gene containing unusual numbers of nonsynonymous polymorphisms in this region in comparison to the chimpanzee, we analyzed the coding sequences (CDS) of genes within 5 Mb upstream to 5 Mb downstream of M003-A06. It was found that M003-A06 was the only gene with an unusually high number of nonsynonymous substitutions (**Figure S1**).

### Two Distinct Allelic Types (H, C) Existing in the Human Population

**Figure 2:**
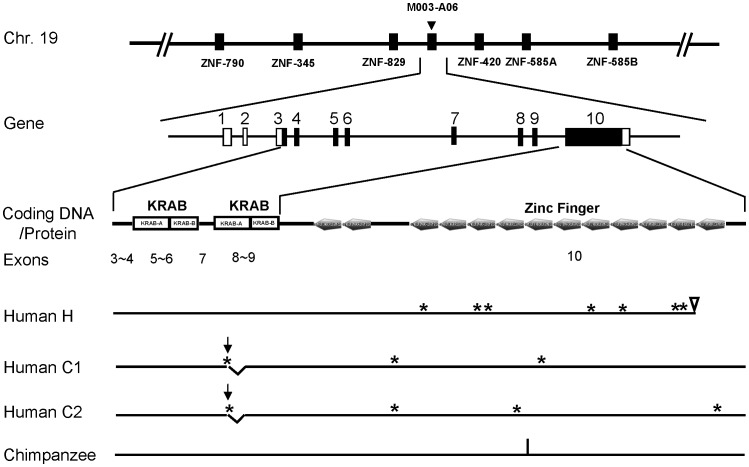
Physical maps of M003-A06 of the human and chimpanzee. Top, chromosomal location of M003-A06 with its flanking genes indicated. Below the top map are the exon-intron gene organization and protein structure of M003-A06. The protein-coding sequences of the exons are in black. The positions of the non-synonymous nucleotides of the human H, C1 and C2 alleles in comparison to the chimpanzee homolog are indicated by the stars. The arrow head and the two arrows indicate the non-synonymous nucleotide substitutions that create the stop codon in the human H allele and the alternative splicing sites in the human C1 and C2 alleles, respectively. The synonymous nucleotide substitution in the chimpanzee gene is indicated by the vertical line.

In the analysis shown in **Figure 1**, most of the non-synonymous differences between human and chimpanzee were found to be located in exon 10. To examine if any of these differences were due to genetic variations in the human and/or chimpanzee populations, we first sequenced the exonic regions of M003-A06 from the genomic DNAs of 11 chimpanzees and 25 Han Chinese. No variation was found in the chimpanzees. In addition, only one synonymous single-nucleotide polymorphism, or SNP, (rs 25756284) was listed in the NCBI chimpanzee genomic SNP database. On the other hand, there existed two major allelic types in the 25 Han Chinese DNA samples (**Figure 2** and **Table 1**). One of them (H-type, or H allele) had the same sequence as that of the reference genome used in the analysis of **Figure 1**. When compared to the chimpanzee, the H allele had one synonymous and seven nonsynonymous changes (indicated by stars, **Figure 2**) in the exon 10 coding region plus a C to T transition (rs1667366) (indicated by arrow head, **Figure 2**), which created a stop codon abolishing the last zinc finger domain. Interestingly, the seven nonsynonymous changes of the H allele (indicated by stars, **Figure 2**) were not present either in the M003-A06 ortholog of gorilla, orangutan or macaque (data not shown), suggesting that the H allele is human-specific as compared to the other primates. The second major allelic type (C-type) contains an A-to-G transition (rs 1667354) in exon 8 that generated an alternative splicing site causing a 51 bp-deletion within the 3' half of the second KRAB-A box in the mature mRNA (**Table 1** and vertical arrows in **Figure 2**). In addition, the coding sequence of the human C allele was similar to chimpanzee and did not contain the seven nonsynonymous changes found in the H type. Nevertheless, there were an additional two or three amino acid changes that subdivided the C type into C1 and C2 subtypes, respectively (**Figure 2** and **Table 1**).

**Table 1:** Summary of the sequence variations in the M003-A06 genes in human populations and chimpanzee.

SNP	Location^a^	Human			Chimpanzee
		H	C1	C2	
rs1667354	479	A (Asp)^b^	G (splicing)	G (splicing)	A (Asp)
rs935706	1039	G (Ala)	A (Thr)	A (Thr)	G (Ala)
rs935707	1130	G (Arg)	A (His)	A (His)	A (His)
rs1667363	1273	A (Ser)	T (Cys)	T (Cys)	T (Cys)
rs1667364	1280	C (Ala)	A (Glu)	A (Glu)	A (Glu)
rs16971886	1382	G (Arg)	G (Arg)	A (His)	G (Arg)
*	1404	T	T	T	C
rs10405238	1462	T (Tyr)	G (Asp)	T (Tyr)	T (Tyr)
rs1345748	1604	G (Cys)	A (Tyr)	A (Tyr)	A (Tyr)
rs1363752	1706	A (Glu)	G (Gly)	G (Gly)	G (Gly)
rs1644698	1879	C (Pro)	G (Ala)	G (Ala)	G (Ala)
rs1363753	1888	G (Gly)	C (Arg)	C (Arg)	C (Arg)
rs1667366	1906	T (Stop)	C (Arg)	C (Arg)	C (Arg)
rs3745770	1972	C (-)	C (Arg)	G (Gly)	C (Arg)

The sequence variations among the H, C1, C2 alleles of human M003-A06 and the chimpanzee M003-A06 gene are listed. The amino acid changes at the non-synonymous SNPs are indicated in the parentheses. The single synonymous nucleotide difference between the human and chimpanzee is indicated by the star.

* The synonymous nucleotide difference between human and chimpanzee.

a The locations of SNPs relative to A (+1) of the start codon (ATG).

b The amino acids at the non-synonymous sites.

Cloning and sequencing of 10 M003-A06 clones from 45 Caucasian testis cDNA libraries confirmed that all three alleles (H, C1 and C2) were indeed expressed in humans. Furthermore, database mining from Hapmap showed that H, C1 and C2 existed in different ethnic groups but with different frequencies. Interestingly, the samples from Japanese or Chinese at Taiwan appeared to have a significantly higher H allele frequency (0.71 in Japanese and 0.72 in Chinese at Taiwan) than those from European (CEU, 0.45) and African (YRI, 0.39) individuals. Although the finding of two distinct allelic types of M003-A06/ZNF568 could be due to the existence of heterologous gene copies, this is an unlikely source of error for three reasons. First, multiple sequences of M003-A06/ZNF568 have been derived by PCR-cloning from the same group of individuals but only two haplotypes have been recovered. Second, the genotype frequencies adhere to Hardy-Weinberg equilibrium (**Table 2**). Finally, this gene does not overlap with any of the previously reported regions with copy number variations [18].

**Table 2:** Allele frequencies and genotype frequencies of M003-A06 in different human ethnic groups.

Ethnic Groups	N	Allele Frequencies			Genotype Frequencies					
		H	C1	C2	HH	HC1	HC2	C1C1	C1C2	C2C2
Taiwanese	1244	0.72	0.17	0.11	0.52 (0.52)	0.24 (0.24)	0.16 (0.16)	0.04 (0.03)	0.03 (0.04)	0.01 (0.01)
JPT	87	0.71	0.19	0.10	0.51 (0.50)	0.27 (0.27)	0.15 (0.14)	0.03 (0.04)	0.03 (0.04)	0.01 (0.01)
CHB	86	0.70	0.20	0.10	0.49 (0.49)	0.29 (0.28)	0.14 (0.14)	0.03 (0.04)	0.05 (0.04)	0 (0.01)
CEU	174	0.45	0.15	0.40	0.21 (0.20)	0.13 (0.14)	0.36 (0.36)	0.02 (0.02)	0.12 (0.12)	0.16 (0.16)
ASW	83	0.42	0.19	0.39	0.15 (0.17)	0.17 (0.16)	0.37 (0.33)	0.02 (0.04)	0.17 (0.15)	0.12 (0.15)
GIH	87	0.40	0.29	0.31	0.13 (0.16)	0.28 (0.23)	0.26 (0.25)	0.06 (0.08)	0.18 (0.18)	0.08 (0.10)
YRI	176	0.39	0.27	0.34	0.18 (0.15)	0.19 (0.21)	0.24 (0.27)	0.07 (0.07)	0.21 (0.18)	0.11 (0.12)

The frequencies of M003-A06 among different ethnic groups except for the Taiwanese are derived from the Hapmap Phase 3 data (http://hapmap.ncbi.nlm.nih.gov). The genotype frequencies expected from the allele frequencies are listed in the parentheses, and all of them are close to the actual genotype frequencies suggesting a Hardy-Weinberg equilibrium (see text). Taiwanese, data from **Figure 6**; JPT, Japanese in Tokyo, Japan; CHB, Han Chinese in Beijing, China; CEU, Utah residents with Northern and Western European ancestry from the CEPH collection; ASW, African ancestry in Southwest USA; GIH, Gujarati Indians in Houston, Texas; YRI, Yoruba in Ibadan, Nigeria.

### Molecular and Cellular Characteristics of M003-A06/Zfp568

#### Expression patterns of M003-A06/Zfp568 in different tissues

**Figure 3:**
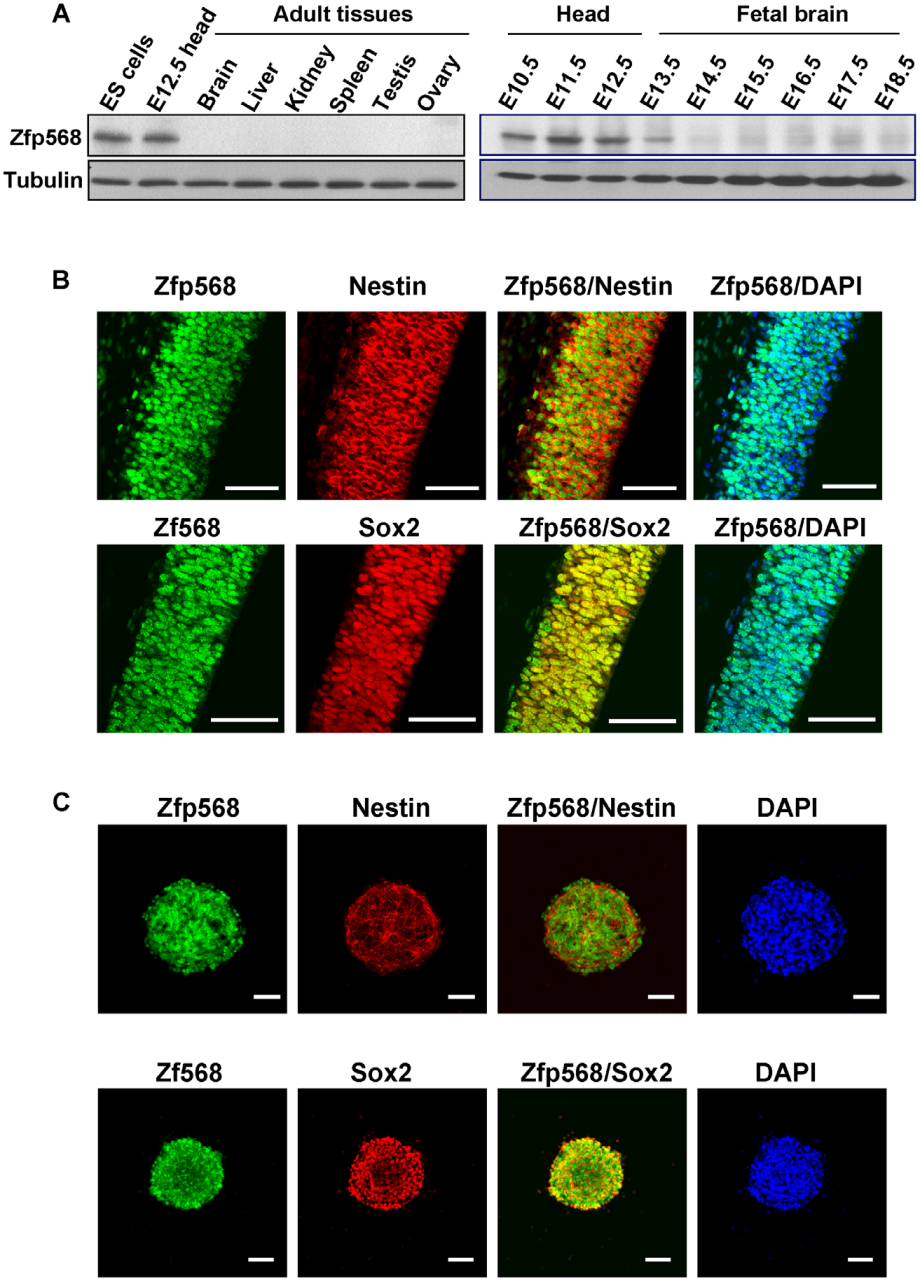
Expression patterns of mouse Zfp568. (**A**) Western blots of total protein extracts from different mouse tissues. The blot of the lysates from the mouse ES cells, E12.5 head, and 6 different adult tissues is shown on the left. The blot of the total protein extracts from the embryonic head (E10.5∼E12.5) and the fetal brain (E13.5∼E18.5) is shown on the right. Tubulin in both blots was used as the loading control. (**B**) Immunofluorescence staining patterns of Zfp568 with the neural stem cells markers Nestin and Sox2 in E12.5 mouse fetal head. The sections were co-stained with the appropriate antibodies. DAPI was used to show the locations of the nuclei. (**C**) Immunofluorescence staining patterns of Zfp568 with Nestin or Sox2 in the neurospheres. Bar, 50 µm.

The DNA sequence identity between M003-A06 and mouse Zfp568 was 74%, indicating that they were evolutionary conserved. Furthermore, mouse Zfp568 also had two KRAB domains in the N-terminal and 11 Zinc-finger domains in the C-terminals respectively, similar to the primate orthologs (**Figure 2** and [17]). M003-A06/Zfp568 appeared to be preferentially expressed in the early fetal brain and in neural stem cells (NSCs). First, quantitative real-time PCR results showed that human M003-A6 mRNA was enriched in the fetal brain relative to other tissues except for the fetal heart (**Figure S2**). Second, Western blotting analysis of the expression profile of Zfp568 in mouse showed that Zfp568 was mainly expressed in the ES cells and E12.5 fetal brain, but was much lower in the adult tissues including the adult brain (left panel, **Figure 3A**). In fetal brains of different developmental stages, the amount of Zfp568 protein was highest between E10.5∼E12.5 and was drastically reduced after E13.5 (right panel, **Figure 3A**).

#### Zfp568 as a neural stem cell marker gene

Since a wave of post-mitotic neurons migrated radially away from the ventricular zone and formed the first layer of the neocortex at E13 [19], the reduced expression of Zfp568 around E13 suggests its importance in neurogenesis during the early mouse brain development. Thus, we examined the spatial expression pattern of Zfp568 in the developing neocortex by immunofluorescence staining of the coronal neocortical sections from E12.5 mouse embryos with different antibodies. As shown, Zfp568 displayed a nuclear staining pattern overlapping with those of Nestin and Sox2, two markers of early neural stem cells, in the cortical layers (**Figure 3B**). A similar pattern of co-expression of Zfp568, Nestin and Sox2 was also detected in the cultured fetal neural stem cells, or neurospheres (**Figure 3C**). Finally, although Western blotting showed little or no Zfp568 expression in the adult mouse brain (**Figure 3A**), immunofluorescence staining result indicated that Zfp568 was expressed in Sox2- and GFAP double-positive cells in the subventricular zone (SVZ) and as well as the subgranular zone (SGZ) of the dentate gyrus (DG) of the hippocampus, two neurogenic regions in the adult mouse brain (**Figures S3A** and **S3B**). As shown, Zfp568 was also expressed in the neurospheres generated from the SVZ of the adult mouse brain (**Figure S3C**). The data in **Figures 3** and **S3** together demonstrated that Zfp568 was expressed in the embryonic stem cells as well as the neural stem cells in both the mouse fetal and adult brains.

**Figure 4:**
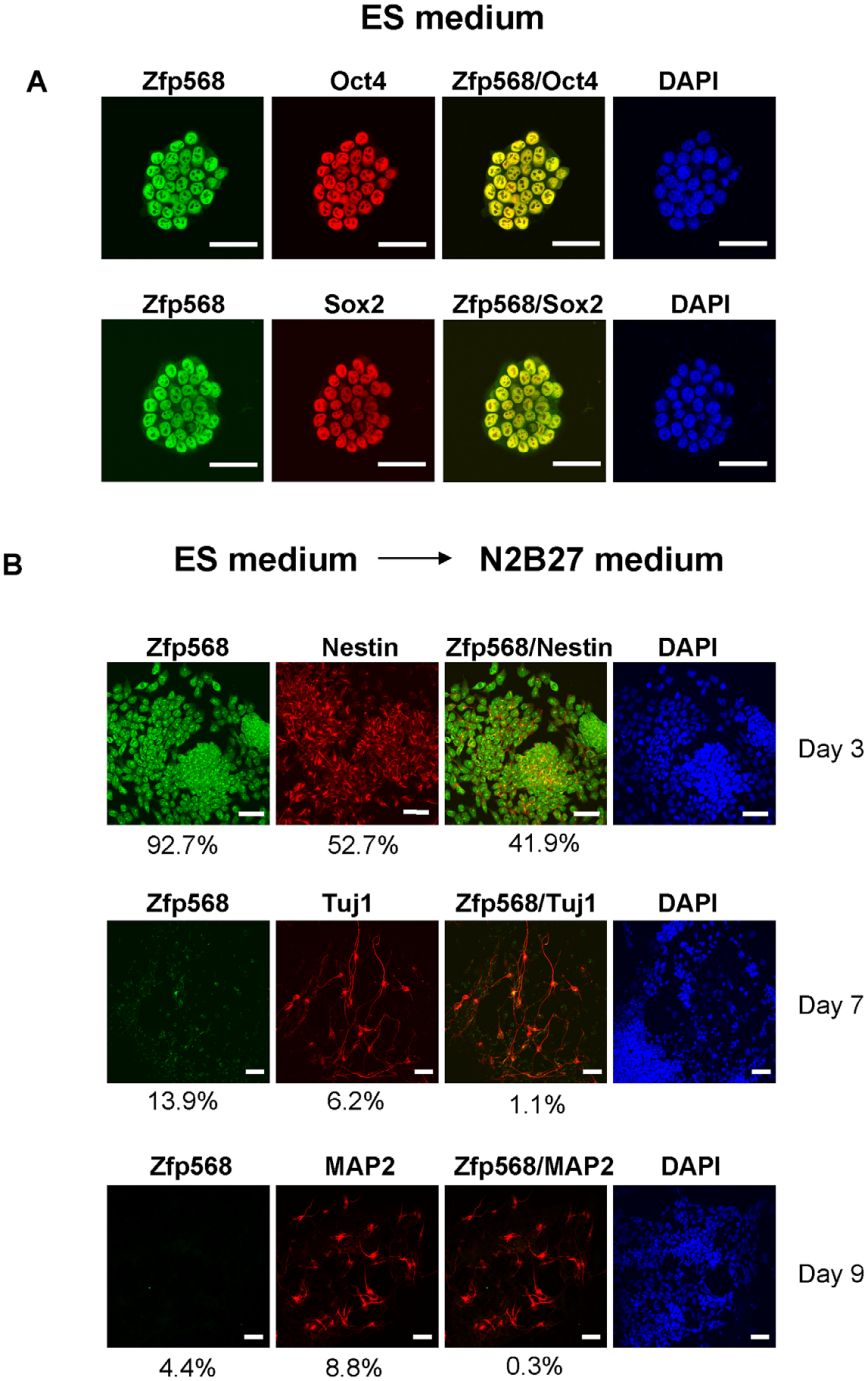
Co-expression of Zfp568 with Nestin but not Tuj1 or MAP2 during neural differentiation of the mouse ES cells. Mouse ES cells were plated at low density in the ES cell medium containing serum and LIF, and then transferred to N2B27 without LIF after overnight incubation (Day 0). (**A**) Co-staining patterns of Zfp568 with the ES cell markers Oct4 and Sox2, respectively, of ES cells in the ES medium. (**B**) Co-staining patterns of Zfp568 with the neural stem cell marker Nestin and neuronal markers, Tuj1 and MAP2, on different days in the N2B27 medium. The percentages of single- and double- stainings, as listed under each panel, were each calculated by scoring the cells in at least three random chosen fields from two independent sets of experiments. (n = 10 for Day3, n = 7 for Day 7, n = 7 for Day 9). Bar, 50 µm.

In view of the above, we examined the expression pattern of Zfp568 at different stages of neural differentiation. Pluripotent mouse ES cells were cultured in ES medium overnight and then switched into N2B27 medium. Under these serum-free monolayer culture conditions, cells expressing the neural stem cell markers, e.g. Nestin, would appear after 3 days. Cells adopting the neural cell morphology and expressing the immature neuronal marker Tuj1 would then show up after 5 days and those expressing MAP2 appeared after 9 days [20], [21]. As seen in **Figure 4A**, Zfp568 was co-stained with the ES cells markers, Oct4 and Sox2, in the ES cell medium. After 3 days in the N2B27 medium, 92.7% of the cells stained positive for Zfp568. Furthermore, more than 41.9% of the Zfp568 signals co-localized with Nestin (first row of panels, **Figure 4B**). Nevertheless, only 1.1% of the cells were positive for both Zfp568 and Tuj1 on day 7 (middle row of panels, **Figure 4B**) and 0.3% of the cells co-stained with anti-Zfp568 and anti-MAP2 on day 9 in the N2B27 medium (bottom row of panels, **Figure 4B**). The immunofluorescence staining data of **Figure 4** indicated that Zfp568/M003-A06 was expressed only in the neural stem cells but not in immature neural progenitors or differentiated neuronal cells.

### Functional Roles of Zfp568 in Early Development of the Mouse Brain and Maintenance/Proliferation of the Neural Stem Cells

**Figure 5:**
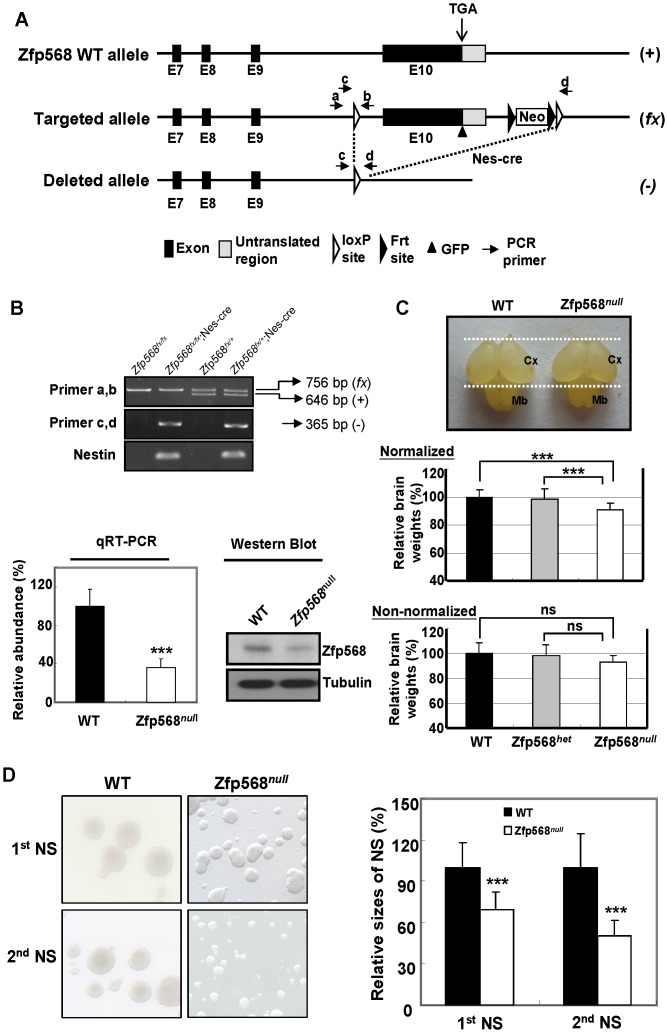
Targeted disruption of the mouse Zfp568 gene in the neural stem cells. (**A**) Targeting strategy. Exon 10 (E10) of Zfp568 was replaced with a “floxed” fragment containing exon 10 followed by an frt-flanking neo cassette. Exon 10 was removed by Nestin-promoter-driven Cre recombinase from the Nes-cre mice. The wild-type, targeted, and deleted alleles are labeled as (+), (*fx*), and (−), respectively, on the right sides of their physical maps. The genotypes of mice carrying the different alleles were validated by PCR (primer sets a/b and c/d) of their genomic DNA. (**B**) Targeted disruption of Zfp568. Top panel, PCR analysis of the offsprings from crosses of the Zfp568*^fx/+^*;Nes-cre male mice with the Zfp568*^fx/fx^* females. Primers a and b were used to differentiate the wild-type (+) and the targeted (*fx*) alleles. Primers c and d were used to detect the deleted fragment as driven by Nes-cre. Lower left panel, quantitative RT-PCR analysis of the level of Zfp568 mRNA in the E12.5 head samples of the wild-type (Zfp568*^fx/+^* or Zfp568*^fx/fx^*). The abundance of the Zfp568 mRNA is relative to that of the GAPDH mRNA. The level of the Zfp568 mRNA from the wild-type E12.5 fetal head is given the value of 100%. Lower right panel, Western blot analysis of the E12.5 fetal head of wild-type (WT) and Zfp568*^fx/fx^*;Nes-cre (Zfp568*^null^*) mice. (**C**) Representative photos of the P0 (postnatal day 0) brains of WT and Zfp568*^null^* (top panel), and the relative P0 brain weights of the WT, the heterozygous Zfp568*^het^* (Zfp568*^fx/+^*;Nes-cre), and Zfp568*^null^* mutant mice (normalized, middle panel; non-normalized, bottom panel). Dashed lines delimit the rostrocaudal extent of the WT cerebral cortex (Cx) and midbrain (Mb). The average brain weight of the WT P0 mice was set as 100%. For the normalized data set (middle panel), the brain weight of each mouse was normalized by its body weight. n = 29 for WT, 15 for Zfp568*^het^*, and 19 for Zfp568*^null^*. ***, p<0.005; ns, not significant. Note that the relatively large sample sizes have overcome the seemingly large standard deviations in the normalized data set (middle panel). Also note that the average of the non-normalized brain weights of the Zfp568*^null^* mice was also smaller than either Zfp568*^het^* or WT (0.065 g in comparison to 0.069 g and 0.070 g, respectively). But these differences were statistically insignificant likely due to the fluctuations of the brain weights in the neonatal mice (P0) of different litters. (**D**) Left panels, photos of the primary (1^st^ NS) and secondary (2^nd^ NS) neurospheres cultured from the WT and Zfp568*^null^* P0 brains. The averages of the diameters of both the primary and secondary neurospheres were calculated and shown in the right panel. The averages of the WT neurospheres (filled bars) are set as 100%; *** p<0.005.

Zfp568 protein, or CHATO, regulated the convergent extension in the mouse embryo and it was also required for the control of morphogenesis of the yolk sac and placenta. In addition, the homozygous null mice died at E9.5–10 [17], [22]. In view of its restricted expression in the fetal head (**Figure 3A**) and NSC (**Figures. 3B–3C** and **4B**), we evaluated whether Zfp568 was an important gene for controlling the early development of the mouse brain. For this, we used a conditional gene-targeting approach to knockout Zfp568 expression in neural stem cells of mice, thus bypassing the early embryonic lethality of the homozygous Zfp568 mutant (**Figure 5A**). Exon 10 of the Zfp568 locus was deleted by crossing the Zfp568*^fx/fx^* mice with mice carrying Nes-cre, which was expressed in the central nervous system (CNS) stem/neural progenitors starting at embryonic day 10.5 (E10.5) [23], [24]. The resulting offsprings (Zfp568*^fx/+^*;Nes-cre) were backcrossed to Zfp568*^fx/fx^* mice thus causing the loss of the Zfp568 locus in the NSCs by E12.5 (**Figure 5A and 5B**). The homozygous Zfp568*^fx/fx^;*Nes-cre mutant mice generated as described above were born in the expected Mendelian ratio, and survived to the adulthood. The Zfp568*^null^* mice were more aggressive upon handling. Furthermore, although breeding of the Zfp568*^null^* females (n = 4, 7 litters) to WT males and vice versa (n = 7, 15 litters) gave viable offspring, the pups were invariantly subjected to infanticide at birth.

Interestingly, the normalized average of the relative brain weights of the Zfp568*^fx/fx^*;Nes-cre mice at birth (postnatal day 0 or P0) was significantly smaller than either the wild type control (91% of the control, p<0.005; **Figure 5C**) or the heterozygous mutant mice (92.6% of the heterozygous mutant, p<0.005; **Figure 5C**). We also performed hematoxylin and eosin (H & E) staining to compare the sizes of different brain subregions of the P0 Zfp568*^fx/fx^*;Nes-cre mice and the wild type controls (**Figure S4A**). It was found that the reduced brain weight of the mutant mice was not due to defects in the cortical layering or neuronal migration. Nor was any specific region(s) of the mutant brain particularly smaller than in the wild type (**Figure S4A**). Relevantly, the average difference between the brain weights of the newborns of the MCPH5/Aspm-null mice and those of the control mice was also relatively small [25]. In contrast to the mice at early development, however, the relative brain weights of the adult WT and Zfp568*^null^* mutant mice were similar (**Figure S5**). Like the P0 mice (**Figure S4A**), H & E staining showed no obvious difference in the subregions of the brain between the Zfp568*^null^* and WT mice (**Figure S4B)**. Also, the mutant mice did not perform better or worse than the WT mice in the Morris water maze test (**Figure S5C**). The data of Figures 5, S4 and S5 suggested that Zfp568/M003-A06 played a role in the early development of the mouse brain.

We also performed neurosphere assay to examine whether Zfp568 plays a role in the maintenance and proliferation of NSCs [24], [26], [27]. In this assay single neural stem cells were allowed to proliferate to form a ball of undifferentiated cells (the neurosphere) and most of the differentiated cells would not be able to survive [26]. Furthermore, the primary neurospheres could be subcultured to form the secondary neurospheres, a measure of the NSC proliferation and self-renewal. Evidence for a role of Zfp568 in the maintenance and proliferation of NSC was corroborated by a progressive loss of the *in vitro* renewal of Zfp568-deleted P0 NSC in the neurosphere culture (**Figure 5D**). As shown, the average sizes of both the primary (1^st^) and secondary (2^nd^) neurospheres grown from NSCs derived from the homozygous Zfp568*^fx/fx^;*Nes-cre mice were 70% and 50%, respectively, of those grown from NSC of the WT mice (**Figure 5D**). RNAi knockdown of Zfp568 also induced cell differentiation of Neuro2A cells in culture (unpublished data). The data in **Figure 5** indicated that Zfp568/M003-A06 contributed to the maintenance and self-renewal of NSC. Furthermore, this function of Zfp568/M003-A06 likely contributed to the early development of the mouse brain.

To explore the possible function of Zfp568 in adult neurogenesis, we examined the *in vivo* proliferations of NSCs in the Zfp568*^null^* mice and WT controls using a saturation BrdU (5′-bromo-2′-deoxyuridine) pulse-labeling method [28], [29] that could label the entire pool of proliferating NSCs within a 12 hr-period. (**Figure S6**). BrdU is a thymidine analog that incorporates into dividing cells during DNA synthesis. Quantitative analysis at 12 hr following the last BrdU injection showed that SVZ of the Zfp568*^null^* mouse brain had ∼30% less BrdU^+^ cells when compared to the WT controls (**Figure S6B**). In contrast, Zfp568*^null^* showed no significant difference in BrdU incorporation in DG of the hippocampus in comparison to WT (**Figure S6C**). Furthermore, the numbers of either the Nestin^+^GFAP^+^ radial glial-like cells or the Nestin^+^GFAP^−^ nonradial glial-like cells in DG [30], [31] that had incorporated BrdU were also similar between the WT and Zfp568*^null^* mice (**Figure S6D)**. In conclusion, the data of **Figure S6** indicated that Zfp568 deficiency led to the reduction of the proliferation rate of NSCs in the adult SVZ but not DG of the hippocampus.

### Genetic Association between M003-A06 and Human Head Size

**Figure 6:**
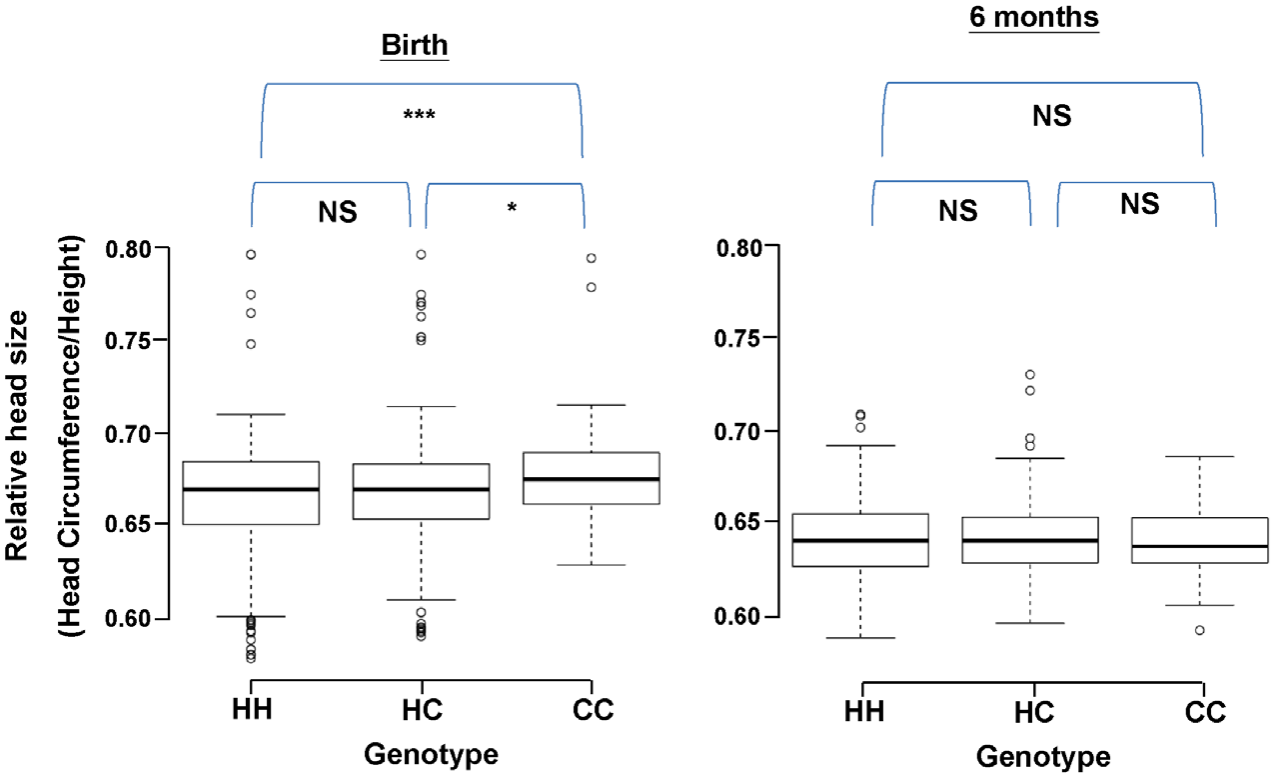
Associations between different genotypes of M003-A06 and the relative head sizes. Associations with the relative head sizes of Chinese at Taiwan. The DNA samples of 1,244 Chinese children at Taiwan were collected at birth and 6 month of age. After genotyping, the combined C allele frequencies (C1+C2) and the relative head sizes were calculated as described in the Materials and Methods and compared (Numbers of the HH, HC and CC are 653, 490 and 101, respectively). Note the significantly larger relative head size for the CC genotype than for either HH or HC at birth (p = 0.0018, left panel), but not at the age of six months (p = 0.8, right panel). *p<0.05, *** p<0.001, NS: not significant.

With the potential functions in neurogenesis described above, the M003-A06 gene could be important for early brain development. We therefore examined the possible association between the different genotypes of M003-A06 and brain/head development in a Taiwanese population of 1,244 unrelated individuals. Since it would be impractical to measure the brain size of every newborn using MRI, we examined the literatures [32], [33] and used head circumference as an index for the brain size of the fetus/newborn. Because the gestational age significantly influences the head size (head circumference) at birth [34], we corrected the head circumferences of the different individuals by their heights. Within the same ethnic group, there were no discernible differences in the head circumference/height at different time periods [35]. We therefore examined the association between the ratios of head circumference/height, or the relative head sizes, and the different genotypes/alleles of M003-A06. Because of their relatively low frequencies in the population of Japanese or Chinese at Taiwan, the C1 and C2 alleles were combined in our analysis as a single allele, the C allele. The average relative head size was significantly larger for the CC genotype than for either HH (*p*<10^−3^) or HC (*p* = 0.012; left panel of **Figure 6**) at birth, but not by six months of age (p = 0.8; right panel of **Figure 6**). After controlling for sex, length of pregnancy, and body weight, individuals with the CC genotype still had larger relative brain sizes than those with the HH or HC genotype at birth (*p* = 0.0018; **Table 3**).

**Table 3:** Analysis of variance (ANOVA) of the relative brain sizes and other parameters at birth.

	Degree of freedom	Sum Square	Mean Square	F value	Pr(>F)
Genotypes*	1	0.007	0.007	9.790	0.002
Length of Pregnancy (week)	1	0.007	0.007	10.235	0.001
Body weight (g)	1	0.000	0.000	0.023	0.880
Sex	1	0.000	0.000	0.498	0.480
Residuals	1238	0.898	0.001		

The genotype CC is compared against HC and HH and a dominant effect is assumed. Among the four variables considered, only the genotype and length of pregnancy in weeks were significant (p value <0.05). The rest of the variables including the body weight in grams and genders, all have p values greater than 0.05, indicating that they were not associated with the relative brain sizes.

* Dominant effect is assumed. The genotype CC is compared with HC and HH.

In order to examine whether this association was unique to the Taiwanese population, we further plotted the frequencies of the H allele against the relative head sizes among five ethnic groups: Chinese at Taiwan, Japanese, Indians, African Americans and European Americans. Remarkably, the H allele frequencies were negatively correlated with the relative head sizes at birth (*p* = 0.018, r = 0.936, left panel of **Figure S7**). The association became insignificant at the age of six months (*p* = 0.725, r = 0.149, right panel of **Figure S7**). Consequently, M003-A06 appeared to be involved in the early development of the human head, with the H alleles associated with a smaller relative head size at infancy.

## Discussion

In this study, we have identified a KRAB-Zn finger protein-encoding gene (M003-A06) that has rapidly evolved in the human lineage since its separation from the chimpanzee and consists of two alleles H and C. Furthermore, the expression of the mouse homologue of M003-A06, Zfp568, is enriched in the early fetal brain and the protein is required for the maintenance of the undifferentiated states and self-renewal of the neural stem cells. Thus, M003-A06/Zfp568 very likely plays a general role in early brain development in vertebrates as well as a specific role in human brain function and development. Indeed, the frequencies of the C/H alleles of M003-A06 appear to be genetically associated with the relative head sizes at birth both within one ethnic group and among different ethnic groups.

In comparison to the human C alleles and the chimpanzee M003-A06 gene, the human H allele has seven out of eight changes located in the zinc-finger domain and a deletion of the last zinc-finger (**Figure 2**), suggesting a functional shift of its DNA-binding specificity [36]. In the KRAB zinc-finger proteins, the KRAB-A box plays a key role in transcriptional repression through binding to co-repressors [37]. Related to this, it has been shown that one point mutation in the KRAB-A box of Zfp568 fails to regulate the convergent extension in mouse embryo and results in the embryonic lethal phenotype [17]. Thus, the lack of the 3' half of the KRAB-A box in the human C1 and C2 alleles of M003-A06/ZNF568 is expected to have an important impact on the regulatory function of the human protein. In summary, since the separation of the human and chimpanzee lineages, the H and C alleles of human M003-A06/ZNF568 have each acquired their functions as the result of drastic and rapid sequence changes in comparison to the chimpanzee ortholog.

Although the direct genotype-phenotype connection of M003-A06/ZNF568 remains to be defined, we suggest that M003-A06/ZNF568/Zfp568 is important for neurogenesis during early brain development. First, M003-A06/ZNF568/Zfp568 expression is likely restricted to the ES cells and NSCs of the early fetal brain, as suggested by the expression pattern of the mouse homolog of M003-A06, Zfp568 (**Figures 3** and **4**). Secondly, Zfp568 ablation in NSCs causes a reduction of the mouse brain size at birth (**Figure 5A–5C**). A smaller brain size could result from reduced mitotic rates, increased cell deaths, changes in cell fate choice, or a combination of these factors. Since Zfp568-depletion in NSCs causes a decrease of the neurosphere size, which becomes more marked with passage from the primary to the secondary neurospheres (**Figure 5D**), M003-A06/ZNF568/Zfp568 is likely one of the genes required for normal NSC maintenance/proliferation which in turn mediates its function in the control of the fetal brain size. Following the above, since the Nes-cre directed knockdown of Zfp568 starts from E10.5 which is before the physiological time (E13.5) of shutdown of Zfp568 expression in the NSC [23], [24], it is expected that NSCs in the mutant mice would begin to differentiate early thus leading to the reduced neuron numbers and consequently the smaller fetal brain.

With respect to the NSC maintenance/proliferation function of Zfp568/M003-A06, it should be noted that several genes have been reported to also function in neurogenesis and/or control of the early brain development [23], [27], [38]. Among those genes, conditional deletion of survivin or αE-catenin, driven by the Nes-cre system, has resulted in postnatal deaths of mice shortly after birth with respiratory insufficiency [38] or with enlarged heads but developmental retardation of the body growth [23], respectively. In contrast, heterozygotes as well as homozygotes with Zfp568 gene-knockout survive to the adult stage and are fertile. Similar to Zfp568, Nes-cre mediated gene knockout of Sox2, which encodes a transcription factor that is also expressed in ES cells and in NSCs at an early stage of the CNS development [24], has resulted in a slight size reduction of the posterior ventrolateral cortex at birth. However, NSCs and neurogenesis were completely lost in the hippocampus leading to dentate gyrus hypoplasia in the Sox2^1oxP/loxP^;Nes–cre mice 7 days after birth [24], a phenotype not exhibited by our Zfp568*^fx/fx^*;Nes-cre mice. Finally, a group of genes named the autosomal recessive primary microcephaly (MCPH) may also be involved in brain size control, since individuals with MCPH gene mutation(s) were born with reduced brain sizes. Only mice with whole body-deficiency of the MCPH homolog(s) have been studied. Among them, the MCPH7/SIL-null mice died *in utero* after embryonic day 10.5 (E10.5) because of a body axis specification defect [39]. The MCPH1/BRIT1-null mice, on the other hand, have a lower birth rate than the normal Mendelian ratio would dictate [40]. Notably, both MCPH3/Cdk5rap2 and MCPH5/Aspm-null mice exhibited microcephaly at birth, and the degrees of reduction of the brain sizes of those mutant mice [25], [41] are comparable to our Zfp568*^fx/fx^*;Nes-cre mice (**Figure 5C**). The positions of M003-A06/ZNF568/Zfp568 in the regulatory networks of NSC maintenance/proliferation and brain size control, respectively, await definition.

Adult neurogenesis is a dynamic, finely tuned process subjected to modulation by various physiological, pathological, and pharmacological stimuli [42]. Interestingly, we have noticed that Zfp568 is expressed in the adult NSCs as well (**Figure S3**). Neurogenesis occurs continuously in two brain regions of the adult rodents, i.e. SVZ of the lateral ventricles and SGZ of the DG in the hippocampus [42], [43]. The Zfp568*^null^* mice have reduced cell proliferation in the SVZ but not in the DG (**Figure S6**). The normal proliferation of NSCs in DG, the neurogenesis within which has been suggested to be involved in spatial memory formation [44], [45], of the adult Zfp568*^null^* mouse brain is consistent with the similar performances of the WT and mutant mice in water maze test (**Figure S5C**). However, the effect of reduced NSC proliferation in SVZ of the mutant mice is unknown at the moment. Finally, we have observed a reduction in the brain weight of the Zfp568*^null^* mice at birth (**Figure 5C**), but the brain size of the mutant mice catches-up in the adult stage (**Figure S5**). Several possibilities could explain for this result. First, gliogenesis mostly occurs postnatally [46]. Thus, a higher rate of gliogenesis after birth may compensate for the smaller brain weight at birth. Second, the average neuronal size in the mutant mice during brain expansion in the early postnatal stage might become larger [47]. Thirdly, stage-specific expression of particular genes may increase the brain weight after birth [48]. Inhibition of apoptosis during the postnatal life in the mutant mice [47], [48] may also account for the observations described in **Figure S5**. It should be noted here that, in interesting parallel to the differential effects of depletion of Zfp568 on the brain size of mice at early development and the adult stage, the presence of the H allele of M003-A06 is associated with a smaller head size at birth but this association disappears among babies of the age 6 months (**Figures 6** and **S7**). Furthermore, database mining has revealed a positive correlation between the adult head sizes among the different ethnic groups and the H allele frequencies of M003-A06 of these groups (**Figure S8**). The molecular and cellular basis of the observed associations of Zfp568 and M003-A06 with the brain/head sizes of mice and human, respectively, await further investigation.

Why would humans preserve two distinct and likely adapted allelic types of the M003-A06/ZNF568 gene within the populations? We propose a tentative scenario to explain these observations in relation to the evolution of M003-A06/ZNF568. That is, the human brain enlarged after its separation from chimpanzee. However, due to certain disadvantages of having larger brains, it was important for humans to acquire new gene(s) or new allele(s), such as the H allele of M003-A06/ZNF568, which could constrain the brain size from increasing further. The effect of M003-A06/ZNF568 on the relative brain size during early infancy, as revealed by the gene knockout studies of **Figure 5**, is supported by our analysis of the head sizes of human newborns presented in **Figure 6**. Interestingly, Montgomery *et al.* have reported a stronger association of MCPH5/Aspm and MCPH3/Cdk5rap2 evolution with the neonatal brain size than with the adult brain size in the anthropoid primates [49]. That result suggests that head size is controlled both genetically and evolutionarily. Notably, despite the obvious advantages of the larger brains [50], they take longer to mature [51], have very high metabolic costs [52], and reduce the efficiency of bipedal locomotion because the pelvic aperture must still allow for birth [53]. With respect to the last point, it has long been acknowledged that the combination in humans of a narrower pelvis necessary for bipedalism and a bigger brain has resulted in many obstetrical problems. Specifically, a smaller pelvis benefits the mother in evolutionary terms in relation to her posture and stability when running, but it is also associated with a higher incidence of both obstructed labor and maternal mortality. In fetal terms, however, it is advantageous for the fetus to have a large head because of improved brain growth. The above situations thus have created a conflict in the maternal/fetal relationship. When compared with Caucasian infants, African infants have shorter average gestational length [54], [55] and more frequent meconium-stained amniotic fluid [56], all of which have been hypothesized to be related to the smaller pelvic sizes of Africans compared to Caucasians. Interestingly, Asian women have even smaller pelvises than the Africans [56] and less pelvic organ mobility than the Caucasians [57]. Further, Taiwanese and Japanese infants have the smallest average of the relative head sizes at birth (**Figure S7**), which is not strongly influenced by environment [58], and this is associated with the higher H allele frequencies of M003-A06/ZNF568 in these two populations (**Table 2** **and Figure S7**). A negative association between the relative infant head size and the H allele frequency is also detectable in a contemporary population of Taiwanese infants (**Figure 6**). Thus, the emergence and maintenance of the rapidly evolving allele (H-allele) of the human M003-A06/ZNF568 gene appear to be positively selected by restriction of the head size during fetal development. After delivery, the pelvic size no longer constrains brain development so that there are no significant differences among the average relative head sizes of the 3 genotypes (HH, HC, CC) at the age of six months (right panels of **Figure 6** and **Figure S7**) and the correlation is even reversed in the adults (**Figure S8**). Although more biological or genetic data are needed to establish this correlation, our results suggest that M003-A06/ZNF568 may be one of the long-sought for genes contributing to human-specific brain development.

In spite of this, we acknowledge the caveats of the data presented here. First, reduction of neonatal brain weight upon gene knockout of either Zfp568 (this study) or MCPH5/Aspm [25] was relatively small albeit significant. This could be due to the regulation of the mammalian brain size by a set of genes, including M003-A06/ZNF568/Zfp568 and MCPH-related genes, the functions of which may be overlapping or degenerate. Second, the difference of the average brain sizes, as measured by the head circumferences, between the HC/HH and CC groups of newborns is also relatively small although statistically significant. This may be due to our choice of one single ethnic cohort for analysis, since the human head size variation is likely neutral or under very weak selection in recent human populations. Thirdly, it should be emphasized that the link between the genotypes of M003-A06 and specific phenotypes, i.e. sizes of the heads and pelvis of different ethnic groups, as deduced by a combined use of contemporary DNA sequencing and database mining, is mainly a genetic association in nature. Future direct genotype-phenotype analysis among the different ethnic groups, as we have done for the Han Chinese newborns, would help to strengthen this link. Future comparison of the proliferation rates of NSC derived from induced pluripotent stem (iPS) cells expressing the human H allele, human C allele, and the chimpanzee ortholog of M003-A06, respectively, would also be a good test of our hypothesis. Finally, transgenic mice studies might help to establish the differential effects of the H and C alleles of M003-A06 on the brain size, although the mouse could be too distant a species from human to test this.

## Materials and Methods

### Sequence Analyses

The 1,668 brain expressed genes used for analysis shown in **Figure 1** were previously defined and deposited in the DNA Data Bank of Japan (http://www.ddbj.nig.ac.jp) under accession number AB170063-174733 [14]. Briefly, human coding sequences (CDSs) were cross-blasted with the chimpanzee and rhesus monkey CDSs. Only genes consistently showing the highest scores and lowest E values in all three-way blast (human-rhesus, chimpanzee-rhesus, and human-chimpanzee) were retained as the putative orthologs. To construct the alignment of the human-chimpanzee-rhesus trios, the CDSs of putative orthologs from the three species were translated and aligned using Clustal W (http://www.clustal.org), and back-translated to their corresponding DNA sequences using TRANALIGN software from the EMBOSS package (http://emboss.sourceforge.net). For genes within the 10 Mb interval centered around M003-A06, the orthologs of the chimpanzee and rhesus monkey were also identified and aligned using the aforementioned method. For each putative pair of the orthologs in human, chimpanzee, and rhesus monkey, the numbers of the synonymous substitutions per synonymous site (Ks) and numbers of the nonsynonymous substitutions per nonsynonymous site (Ka) were calculated using the codeml implemented in Phylogenetic Analysis by Maximum Likelihood (PAML) software.

For haplotypes comparison among different human populations, haplotypes data of 6 human populations were downloaded from the HapMap web site (http://hapmap.ncbi.nlm.nih.gov/downloads/phasing/2009-02_phaseIII/HapMap3_r2/).

### Genomic DNA Isolation, PCR Amplification and DNA Sequencing

The genomic DNAs of the Caucasians and blacks were from the Coriell Cell Repositories: 10 Northern Europeans, Human Variation Panel HD01; 10 Italians, Human Variation Panel HD21; 10 African Americans, Human Variation Panel HD04. Genomic DNA was isolated using the DNA Blood kit from Chemagen. 25 of the 1,244 samples were used for the analysis in **Table 2**. The human cDNAs were from the Human MTC™ Panel (Clontech). PCR reactions were carried out using Advantage 2 PCR kits (Clontech). The sequences of the primers used for amplification of different regions are available upon request. Cycle sequencing was done with the ABI PRISM BigDye terminator Sequencing Kit (Applied Biosystems). DNA sequencing was carried out using the ABI 3730 DNA analyzer (Applied Biosystems).

### Ethics Statement

The genomic DNAs of 1,244 Chinese at Taiwan were isolated from heel blood samples at the National Taiwan University Hospital (NTUH) and the blood donors provided written parental informed consent was obtained using forms approved by the National Taiwan University Hospital (NTUH) Research Ethics Committee (REC) (IRB:200905039R). Parental informed consent was obtained from the parents/guardians of all children involved in the study.

### Data Measurement of Infants

The children were all born in NTUH during 2009–2010 and were examined at birth and 6 months. Measurements were made under standardized conditions. Briefly, a non-stretchable plastic tape measure was placed around the head at the same level on each side, crossing the forehead superior to the supraorbital ridges and passing the prominence of the occiput, for the measurement of the head circumference. The children were weighed naked (in a bowl) on steelyard platform scales. The length was measured on a custom built board with a fixed steel measure. The genders and the length of pregnancy were also recorded. All of the measurements were blinded to the genotypes by the trained nurses in NTUH.

### Cell Cultures

#### ES cells

E14TG2 (ATCC number: CRL-1821), a mouse cell line adapted to feeder-free conditions, was cultured and maintained following procedures as described [59]. Briefly, all the cells were maintained on gelatin-coated dishes in Dulbecco’s modified Eagle’s medium (DMEM; Gibco) supplemented with 15% heat-inactivated fetal bovine serum (FBS; Gibco), 0.055 mM 2–mercaptoethanol (Gibco), 2 mM L-glutamine, 0.1 mM MEM non-essential amino acid, 5000 U/mL penicillin/streptomycin, and 1000 U/mL Leukemia Inhibitor Factor (LIF; Chemicon).

Neural differentiation of the ES cells under spreading-culture conditions was performed as described [20]. Briefly, ES cells were cultured on gelatin-coated dishes in ES cell medium overnight and the switched into the N2B27 medium (1∶1 mix of DMEM/F12 supplemented with modified N2 and neurobasal medium supplemented with B27; Invitrogen) at a concentration of 2×10^4^ cells/cm^2^, with the medium renewed every 2 days.

#### Neurosphere assay

The neurosphere cultures from the wild-type (WT) and Zfp568*^null^* (Zfp568*^fx/fx^*; Nes-cre) mice were prepared as described previously [26]. Briefly, the forebrains of the P0 mice or the periventricular regions of the adult (8-week) mice brain were dissected and dissociated mechanically. The dissociated cortical cells (20,000 cells per ml) were cultured on uncoated plates for 5–7 days in serum-free medium containing EGF and FGF2 (20 ng/ml each; Invitrogen) following the typical protocols of neurosphere growth [60]. Cells from the primary neurospheres were then replated as for the secondary neurospheres. The diameters of both the primary and secondary neurospheres (200∼300 each) were measured after 6 days of plating. All experiments were done in duplicate.

The mouse neuroblastoma cell line Neuro2A (ATCC clone number CCL-131) was maintained in Eagle’s minimal essential medium MEM containing 10% FBS and 1% penicillin/streptomycin in an incubator at 37°C with 5% CO_2_.

### Neuro2A Transfection

Two hours prior to siRNA oligo transfection, fresh medium was added to the culture. The cells were then transfected with either 100 nM siRNA oligo (Si) or scrambled control oligo (Sc) using Lipofectamine 2000 (Invitrogen) according to the manufacturer’s instructions. The transfected cultures were harvested 72 hrs after siRNA/scRNA addition for RNA isolation and morphological analysis.

For the above, the siRNA duplex oligo, 5′-GAGAAAAGUCAGAAAACGUUU-3′, was designed by Dharmacon to target the coding sequence of the Zfp568 mRNA (Si). The scrambled RNAi oligo (Sc), 5′-GAAUAAGAAGCGACAGUAAUU-3′, was used as a control.

### Antibodies

Home-made Zfp568 antiserum was generated by boosting the rabbits with the peptide GRGSELSTHQKIHTGEKPY corresponding to the region from a.a. 625 to 643 of the mouse Zfp568. The antibody was then purified from the sera with an affinity column, concentrated, and stored at −20°C before use. The home-made anti-Zfp568 antibody was specific since (1) no signal could be detected in the ventricular zone of E12.5 head with use of pre-immune rabbit serum and 2^nd^ antibody (**Figure S9A**); (2) siRNA knockdown of Zfp568 in Neuro2A cells was accompanied with reduction of the amount of Zfp568 as detected by this antibody (**Figure S9B**). Anti-tubulin and anti-MAP2 mouse monoclonal antibodies were from Sigma. Anti-Nestin mouse antibody MAB353 was from Chemicon. Anti-Oct4 and Anti-Sox2 antibodies were from Santa Cruz. Anti-Tuj1 antibody was from GeneTex. Anti-BrdU and Anti-GFAP antibodies were from Abcam.

### Western Blotting Analysis

Western blotting was carried out following the standard protocols. Different mouse tissues including dissected embryonic/fetal brains were homogenized and lysed in RIPA buffer (50 mM Tris-HCl, pH 7.5, 150 mM NaCl, 1% NP-40, 0.5% sodium deoxylate, 0.1% SDS, 2 mM EDTA). Total protein (20 µg) was electrophoresed on a 10% SDS polyacrylamide gel, transferred to a PVDF membrane, incubated with primary antibodies overnight, and then with secondary antibodies. The labeled bands were identified using the enhanced chemiluminescence (ECL) detection system (Amersham Biosciences).

### Immunofluorescence Staining

Immunofluorescence staining of the mouse fetal head sections followed standard procedures [24]. The sections were incubated overnight at 4°C with the primary antibodies, and then 1 hr at 20–25°C with the secondary antibodies conjugated with appropriate fluorochromes (Molecular Probes). Staining of DNA was carried out using DAPI (4′,6′ diamidino-2-phenylindole; Molecular Probes).

For immunofluorescence staining of the ES cells and neurospheres, the cells were fixed in 4% paraformaldehyde (PFA) following standard procedures. They were then incubated with the first antibodies overnight at 4°C, washed and incubated with Alexa Fluor 488 goat anti-rabbit IgG or Alexa Fluor 546 goat anti-mouse IgG (Molecular probes). Staining of DNA was carried out using DAPI. For confocal laser scanning microscopy, we used the Zeiss LSM 510 Meta with Axiovision software (Zeiss, Germany). For immunofluorescence staining of the neurospheres, the same protocol was used except that the cells were transferred to the chamber slides one day before staining.

For immunofluorescence staining of the adult mouse brain (4∼6 months), mice were euthanized and perfused transcardially in PBS with 4% PFA. The brain was removed and then immersed in 4% PFA overnight, dehydrated and paraffin embedded. The paraffin embedded brain was sectioned by the vibratome into 5 µm slices. The antigen retrieval and immunofluorescence staining followed the standard procedures.

### Quantitative Real-time PCR Analysis

Human cDNAs from the Human Fetal MTC™ Panel (Cat. No. 636747, Clontech) and Human MTC™ Panel I (Cat. No. K1420-1, Clontech) were used. The mRNA levels were measured by real-time PCR analysis based on SYBR Green detection with the ABI Prism 7500 machine (Applied Biosystems). The results were normalized to GAPDH mRNA. For all the primers used, each pair gave rise to a single product of the expected size, as confirmed by agarose gel electrophoresis and dissociation curve analysis (data are available upon request).

### Conditional Gene Targeting in Mice

Experimental procedures involving animals were approved by the Animal Care and Use Committee of the Institute of Molecular Biology, Academia Sinica with permit number RMiIMBSJ2010057, and all the experimental procedures were performed according to the guidelines established by the Animal Care and Use Committee of the Institute of Molecular Biology, Academia Sinica, Taipei, Taiwan. C57BL/6J mice were used throughout this study.

The removal of the genomic regions was achieved by standard gene-targeting approach using the Cre-lox recombination system. The targeting vector (**Figure 5A**) was generated for deletion of exon 10 of *Zfp568* in the BAC clone RP23-419L22 (Invitrogen) using the Counter-Selection BAC Modification kit (Gene Bridges). A loxP site was inserted into the intron 9 of *Zfp568*. Two frt sites flanking the PGK-neo cassette followed by another loxP site was inserted behind exon 10. To achieve the homologous recombination, the targeting vector was electroporated into C57BL/6 ES cells. G418-resistant clones were genotyped by PCR using an intensity comparison method. Two independent targeted ES cell clones were expanded and microinjected into C57BL/6-C2J blastocysts to generate the chimeric mice, which were then mated with the wild-type C57BL/6-C2J albino mice to obtain mice carrying the Zfp568*^fx^* allele. Male Nes-cre mice carrying the cre-recombinase gene under the control of the nestin promoter and nervous system-specific enhancer [B6.Cg-Tg(Nes-cre)1Kln/J; stock no. 003771; The Jackson Laboratory, Bar Harbor, ME] were crossed with female Zfp568*^fx/+^*. The resulting offspring (Zfp568*^fx/+^*;Nes-cre) male mice were crossed with Zfp568*^fx/fx^* females to obtain the Zfp568*^null^* (Zfp568*^fx/fx^*;Nes-cre) mutant mice. Genomic DNA from the tails was isolated for genotyping by PCR with different DNA primer sets. The primer sequences are available upon request.

### Weight Measurements of the Mouse Bodies and Brains

P0 and adult mice (4∼6 months) mice were obtained from crosses of the Zfp568*^fx/+^*;Nes-cre male mice with Zfp568*^fx/fx^* females. For each P0 mouse, the body weight was measured followed by measurement of brain weight immediately after dissection. Litters of fewer than 6 pups were excluded from this analysis. We normalized the P0 brain weight, but not the adult brain weight, to the body weight because these two factors are highly correlated at birth till postnatal day 23 (r = 0.97) [61].

### *In Vivo* Cell Proliferation

Adult mice (4∼6 months) were given four injections of BrdU (50 mg/kg) within 12 hr to label all dividing cells in adult germinal zones within this time period based on a published paradigm [28], [29], [31]. Mice were then euthanized and perfused transcardially in PBS with 4% PFA at 12 hr following the final BrdU injection. The brain was removed and then immersed in 4% PFA overnight, dehydrated and paraffin embedded. The paraffin embedded brain was sectioned by vibratome into 5 µm slices. The DNA denaturation, antigen retrieval, immunofluorescence staining and quantification of BrdU^+^ cells were followed the procedure described previously.

### H&E Histological Analysis

Brain tissues of the WT and Zfp568*^null^* mice (P0 and 6 month-old of age) were dissected and fixed as described above. 5 µm thick paraffin sections were deparaffinized, rehydrated, and stained with hematoxylin and eosin.

#### Morris water maze task

For spatial learning test, the Morris water maze task was performed as described previously [62], [63]. The animals (4∼6 months) were subjected to four trials per session and two sessions a day, with one session given in the morning and the other given in the afternoon. For a complete test, a total of 6 sessions in 3 days were given. The time spent by the individual mice to reach the platform in the water was recorded as the escape latency.

## Supporting Information

Figure S1 **Plot of the excess of nonsynonymous substitutions vs. Ka for the 10 Mb genomic regions surrounding the human/chimpanzee M003-A06.** The numbers of excess nonsynonymous substitutions (Y-axis) for genes in the regions 5 Mb upstream to 5 Mb downstream of the human/chimpanzee M003-A06 genes and the Ka values were estimated by the maximum likelihood method implemented in PAML and plotted. Note that among all the genes compared, M003-A06 (the open square) has the highest number of excess nonsynonymous substitutions in the human lineage.
(TIFF)

Figure S2 **Expression patterns of M003-A06 in different human tissues.** The levels of M003-A06 mRNAs in different human tissues were compared by quantitative RT-PCR analysis. Eight human fetal and three adult tissue cDNAs were used.
(TIFF)

Figure S3 **Expression of Zfp568 in the adult mouse neural stem cells.** Immunofluorescence co-staining patterns of Zfp568 with the neural stem cell markers in the adult SVZ (**A**), DG of the hippocampus (**B**), and neurospheres (**C**) with use of anti-Zfp568, anti-Sox2, anti-GFAP, and DAPI. The neurospheres were prepared from the periventricular region of the adult mouse brain. SVZ, subventricular zone; DG, dentate gyrus; LV, lateral ventricle; Hil, Hilus; SGZ, subgranular zone; GCL, granule cell layer. Bars, 20 µm (A and B) and 50 µm (C).
(TIF)

Figure S4 **H & E staining of the brains of P0 and adult mice.** The coronal sections of the P0 (**A**) and adult (**B**) brains of the WT and Zfp568*^null^* mice were stained with hematoxylin (H) & eosin (E). Bars, 1 mm.
(TIF)

Figure S5 **Comparisons of the adult brain weights and learning/memory capabilities of the WT and Zfp568** ***^null^*** **mice.** (**A**) Representative photos of the adult brains of the WT and Zfp568*^null^* mutant mice. (**B**) The relative brain weights of the WT and Zfp568*^null^* mutant mice. The average brain weight of the adult WT mice was set as 100%. ns, not significant. (**C**) Morris water maze test results of the adult WT and Zfp568*^null^* mice. The learning/memory capabilities are expressed as the latencies exhibited in six consecutive sessions of the test. Results represent the mean ± SEM (n = 11 for WT and n = 5 for Zfp568*^null^*).
(TIF)

Figure S6 **Effects of Zfp568 deficiency on proliferation of the neural stem cells (NSC) in the Zfp568** ***^null^*** **mouse brains. (A)** Experimental scheme for assessing the neural stem cell proliferation in the adult mouse brains by BrdU labelling. **(B, C)** Immunofluorescence staining patterns of SVZ (B) and DG (C) of the WT and Zfp568*^null^* mouse brain sections with DAPI and antibody against BrdU. n = 4 for each set of samples; ***, p<0.005; ns, not significant. (**D**) Left, representative immunofluorescence co-staining patterns of DG with use of anti-Nestin, anti-GFAP, anti-BrdU, and DAPI. Arrow heads, Nestin^+^GFAP^+^ cells. Arrows, Nestin^+^GFAP^−^ cells. The quantitative analysis is shown in the 2 histograms on the right. For each animal, 10 coronal sections were analyzed. n = 4 mice for WT and Zfp568*^null^*, respectively. Results represent the mean ± SEM. p = 0.376 and 0.671 for the two histograms, respectively. Bars, 100 µm (B and C) and 50 µm (D).
(TIF)

Figure S7 **Associations between the H allele frequencies of M003-A06 and the relative head sizes** **of newborns among different ethnic groups.** The relative head sizes of five ethnic groups were plotted against the frequencies of their H alleles. The H allele frequencies were extracted from the HapMap database. The head and height data were from the following sources: Japanese (open diamond), data from [35]; Chinese at Taiwan (closed diamond), data from **Figure 6**; Indians (closed circle), data from [64]; African Americans (stippled diamond), data from [65]; European Americans (open circle), data from [66]. Note the negative associations of the relative head sizes with the H allele frequencies at birth (p = 0.018, left panel), but not at the age of six months (p = 0.351, right panel).
(TIF)

Figure S8 **Associations between the H allele frequencies of M003-A06 and the relative head sizes** **of adult males** **among different ethnic groups.** The relative head sizes of five ethnic groups are plotted against the frequencies of their H alleles. The H allele frequencies were extracted from the HapMap database. The head and height data were from the following sources: Japanese (open diamond), data from [35]; Chinese (closed diamond), data from http://www.hk-doctor.com/tool/html/TOC_E.htm; Indians (closed circle), data from [67]; African Americans (stippled diamond), data from [68]; European Americans (open circle), data from [35]. *, the data of 17-year old African Americans were used for the analysis. For the other 4 groups, those of the 18-year old males were used. Note the positive associations of the H allele frequencies with the relative head sizes of the 18-year old males (p = 0.018; this figure) as well as 18-year old females (data not shown).
(TIF)

Figure S9 **Specificity tests of the anti-Zfp568 antibody by immunofluorescence staining (A) and by Western blotting (B).** (**A**) Co-staining patterns of the E12.5 brain sections with anti-Zfp568, the pre-immune rabbit serum (pre-immune), or the second antibody (Donkey anti-rabbit 488, 2^nd^ Ab) with DAPI. Note the lack of signal from use of the pre-immune antibody (middle row) and the 2^nd^ Ab (bottom row). Bars, 50 µm (**B**) Western blotting analysis, with use of anti-Zfp568, of extracts from Neuro2A cells transfected with either a scrambled control siRNA oligo (Sc) or a Zfp568-specific siRNA oligo (Si). Duplicated samples were used in the blottings. Tubulin was used as the loading control.
(TIF)
