# Supplementary figures and images for: Targeted Disruption in Mice of a Neural Stem Cell-Maintaining, KRAB-Zn Finger-Encoding Gene That Has Rapidly Evolved in the Human Lineage
Source: PLoS One. 2012 Oct 10;7(10):e47481. doi: 10.1371/journal.pone.0047481 (PMC3468564; doi:10.1371/journal.pone.0047481)

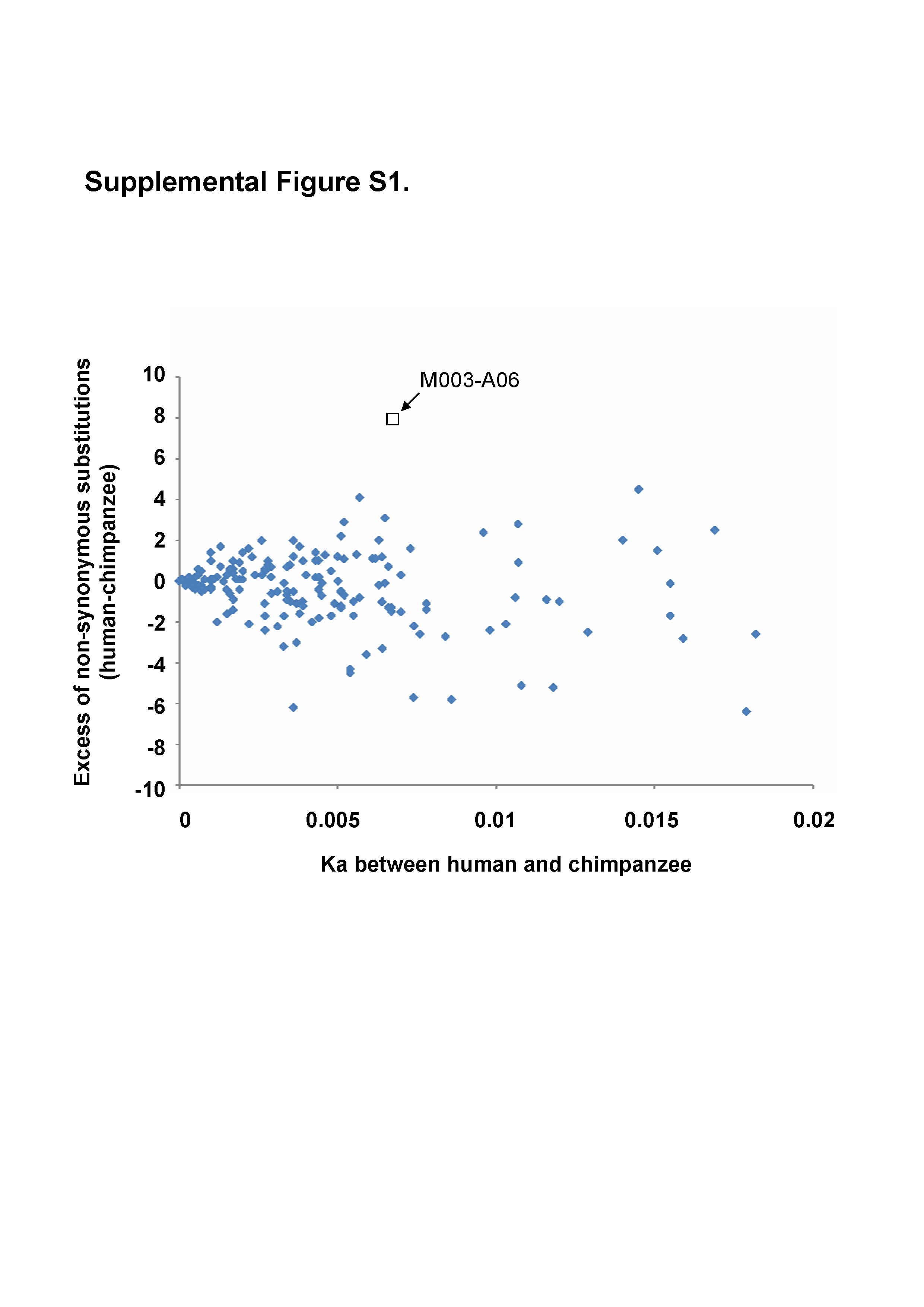

Supplement: Figure S1 — Plot of the excess of nonsynonymous substitutions vs. Ka for the 10 Mb genomic regions surrounding the human/chimpanzee M003-A06. The numbers of excess nonsynonymous substitutions (Y-axis) for genes in the regions 5 Mb upstream to 5 Mb downstream of the human/chimpanzee M003-A06 genes and the Ka values were estimated by the maximum likelihood method implemented in PAML and plotted. Note that among all the genes compared, M003-A06 (the open square) has the highest number of excess nonsynonymous substitutions in the human lineage. (TIFF) [file pone.0047481.s001.tiff]

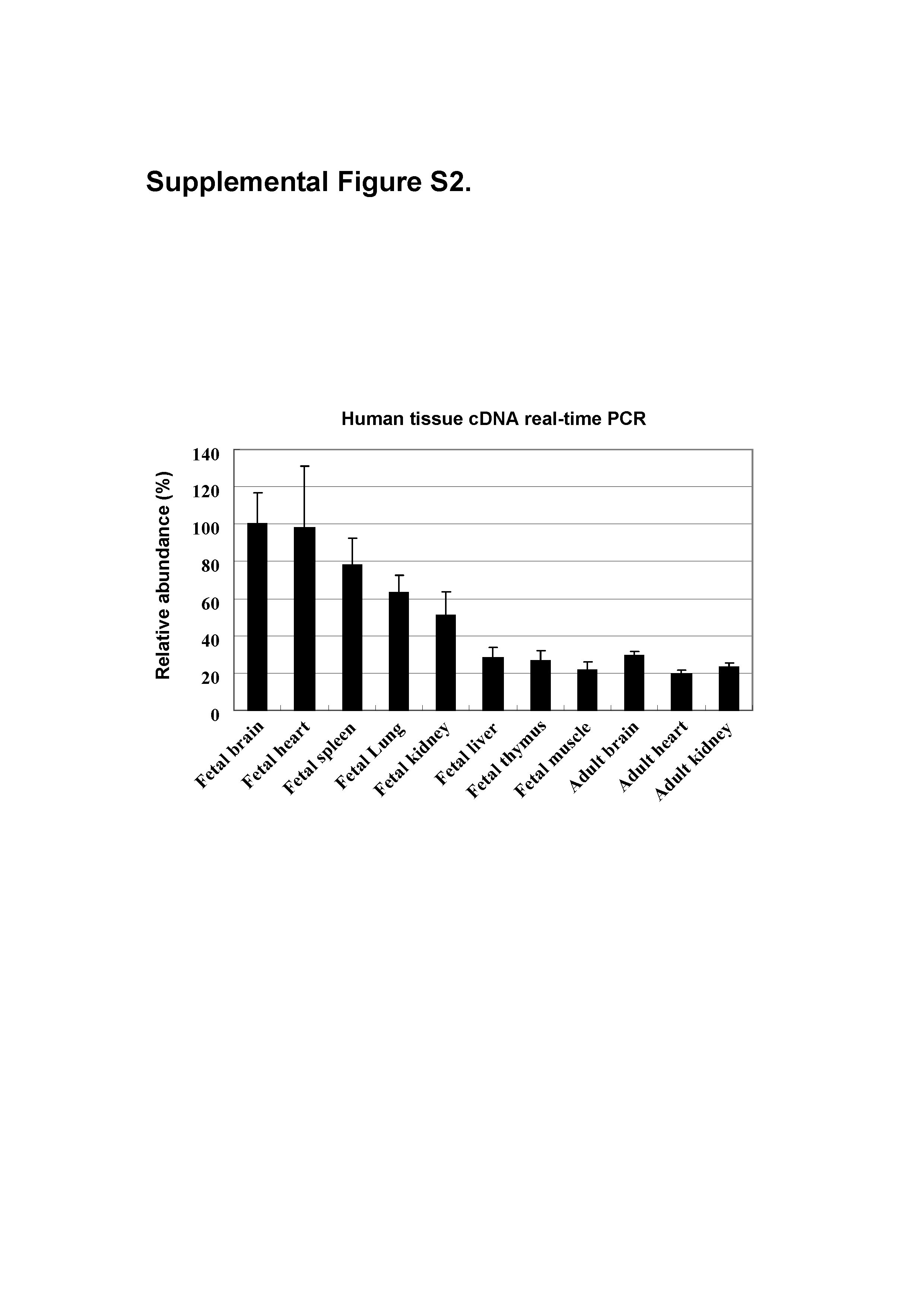

Supplement: Figure S2 — Expression patterns of M003-A06 in different human tissues. The levels of M003-A06 mRNAs in different human tissues were compared by quantitative RT-PCR analysis. Eight human fetal and three adult tissue cDNAs were used. (TIFF) [file pone.0047481.s002.tiff]

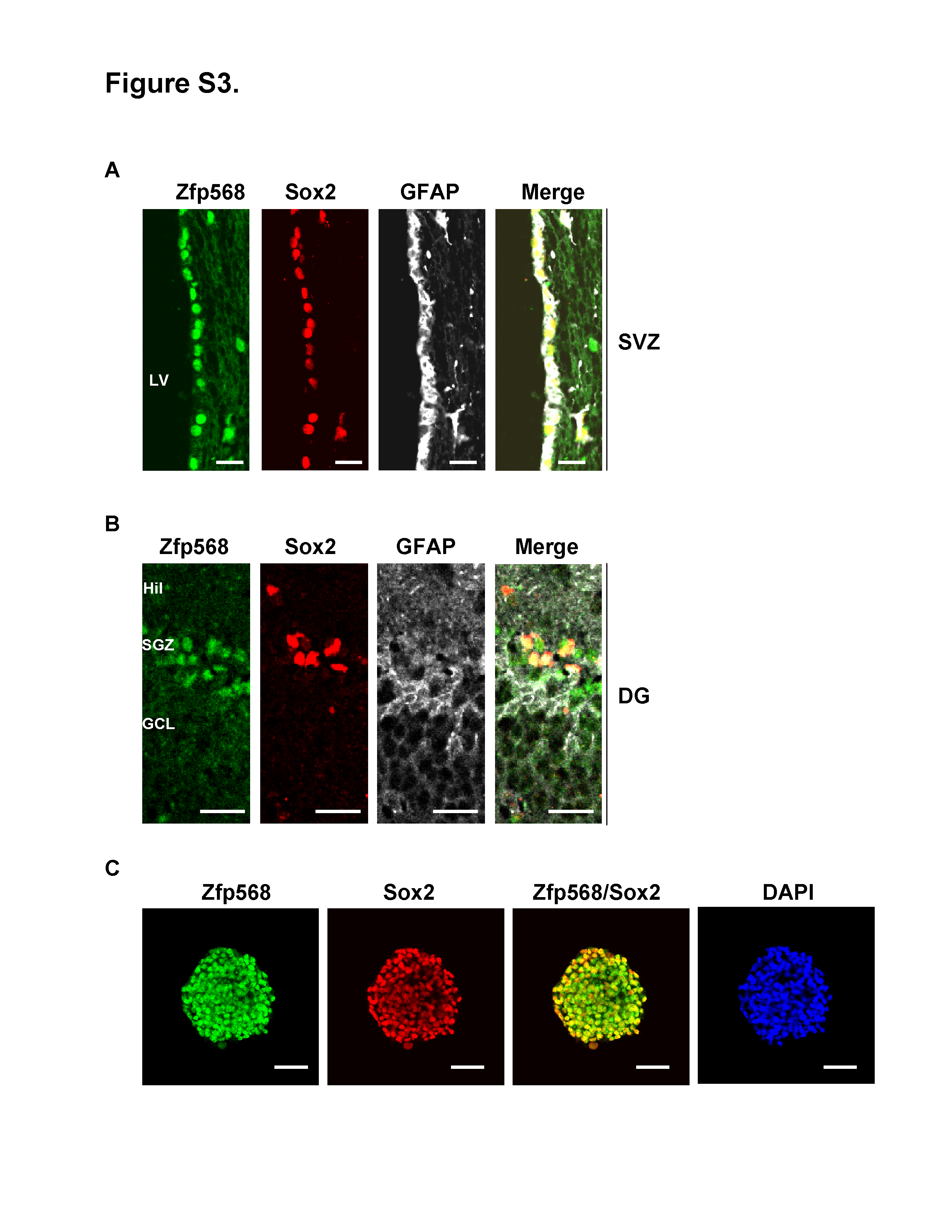

Supplement: Figure S3 — Expression of Zfp568 in the adult mouse neural stem cells. Immunofluorescence co-staining patterns of Zfp568 with the neural stem cell markers in the adult SVZ (A), DG of the hippocampus (B), and neurospheres (C) with use of anti-Zfp568, anti-Sox2, anti-GFAP, and DAPI. The neurospheres were prepared from the periventricular region of the adult mouse brain. SVZ, subventricular zone; DG, dentate gyrus; LV, lateral ventricle; Hil, Hilus; SGZ, subgranular zone; GCL, granule cell layer. Bars, 20 µm (A and B) and 50 µm (C). (TIF) [file pone.0047481.s003.tif]

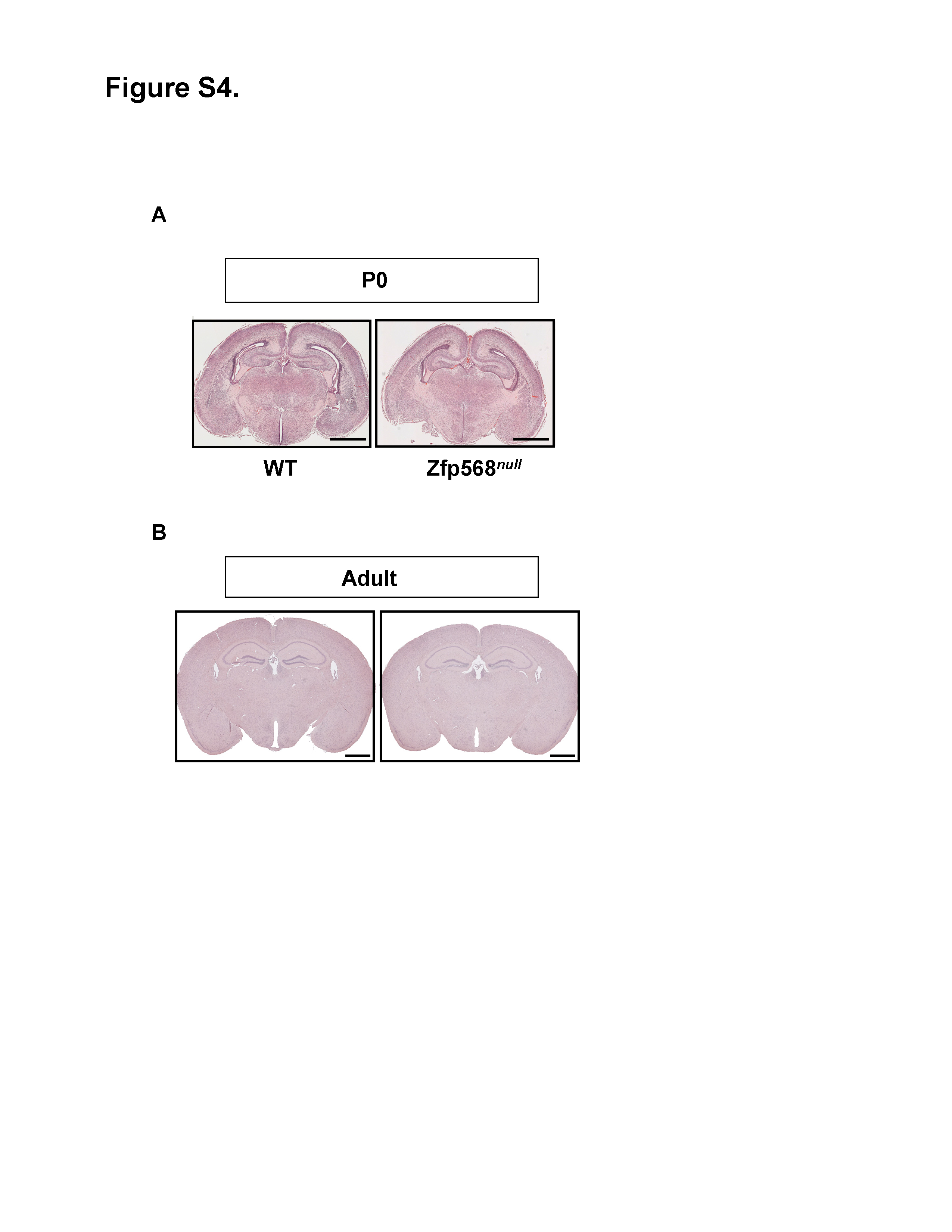

Supplement: Figure S4 — H & E staining of the brains of P0 and adult mice. The coronal sections of the P0 (A) and adult (B) brains of the WT and Zfp568null mice were stained with hematoxylin (H) & eosin (E). Bars, 1 mm. (TIF) [file pone.0047481.s004.tif]

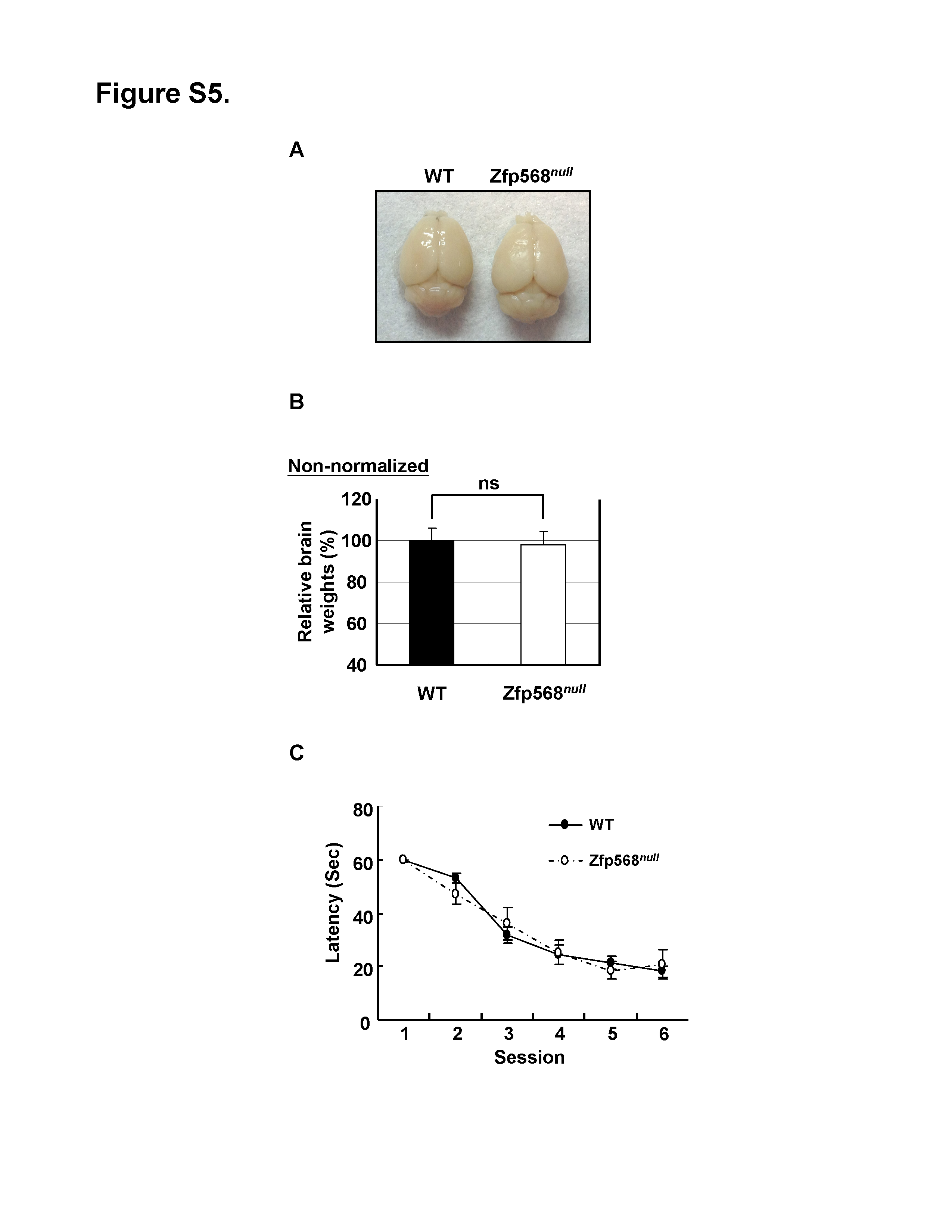

Supplement: Figure S5 — Comparisons of the adult brain weights and learning/memory capabilities of the WT and Zfp568 null mice. (A) Representative photos of the adult brains of the WT and Zfp568null mutant mice. (B) The relative brain weights of the WT and Zfp568null mutant mice. The average brain weight of the adult WT mice was set as 100%. ns, not significant. (C) Morris water maze test results of the adult WT and Zfp568null mice. The learning/memory capabilities are expressed as the latencies exhibited in six consecutive sessions of the test. Results represent the mean ± SEM (n = 11 for WT and n = 5 for Zfp568null). (TIF) [file pone.0047481.s005.tif]

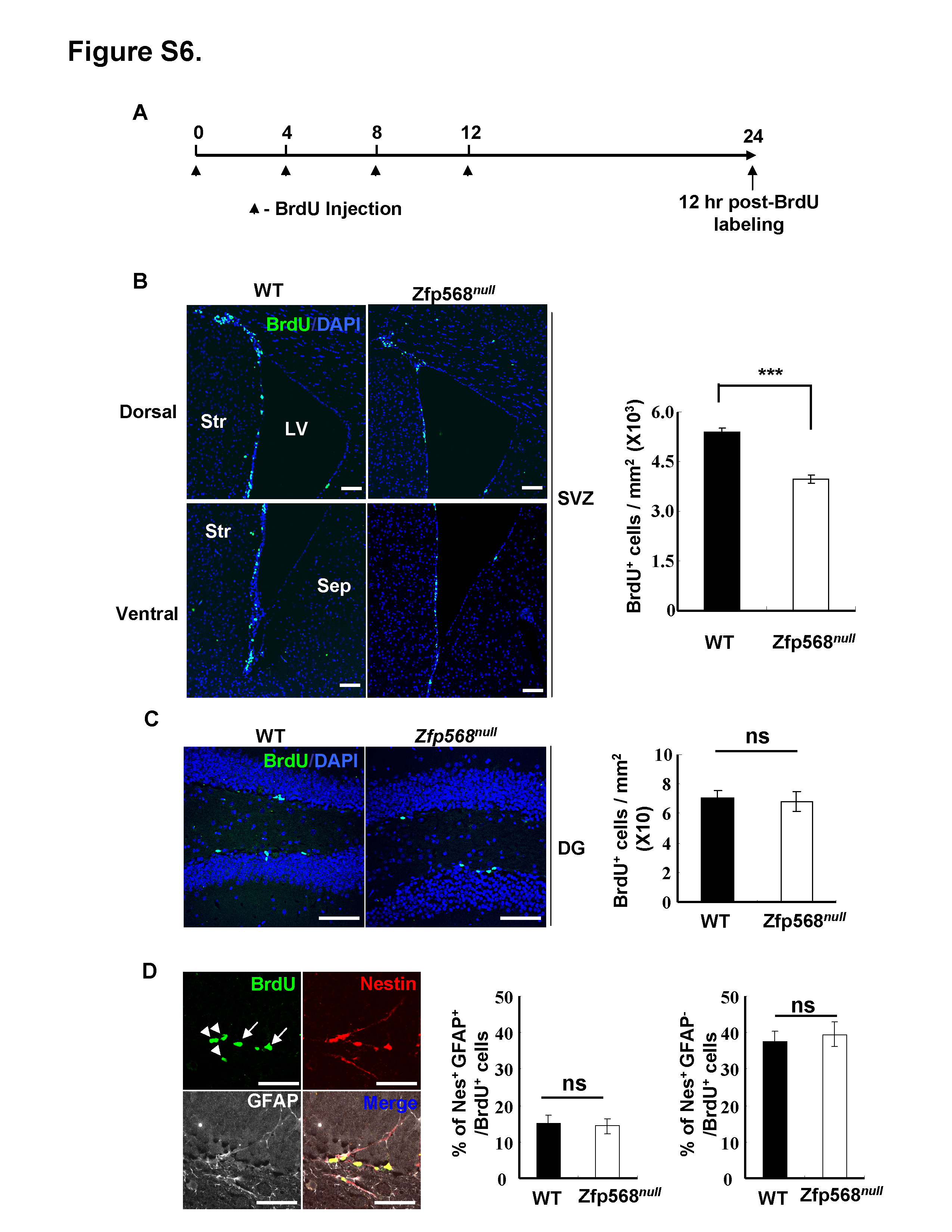

Supplement: Figure S6 — Effects of Zfp568 deficiency on proliferation of the neural stem cells (NSC) in the Zfp568 null mouse brains. (A) Experimental scheme for assessing the neural stem cell proliferation in the adult mouse brains by BrdU labelling. (B, C) Immunofluorescence staining patterns of SVZ (B) and DG (C) of the WT and Zfp568null mouse brain sections with DAPI and antibody against BrdU. n = 4 for each set of samples; ***, p<0.005; ns, not significant. (D) Left, representative immunofluorescence co-staining patterns of DG with use of anti-Nestin, anti-GFAP, anti-BrdU, and DAPI. Arrow heads, Nestin+GFAP+ cells. Arrows, Nestin+GFAP− cells. The quantitative analysis is shown in the 2 histograms on the right. For each animal, 10 coronal sections were analyzed. n = 4 mice for WT and Zfp568null, respectively. Results represent the mean ± SEM. p = 0.376 and 0.671 for the two histograms, respectively. Bars, 100 µm (B and C) and 50 µm (D). (TIF) [file pone.0047481.s006.tif]

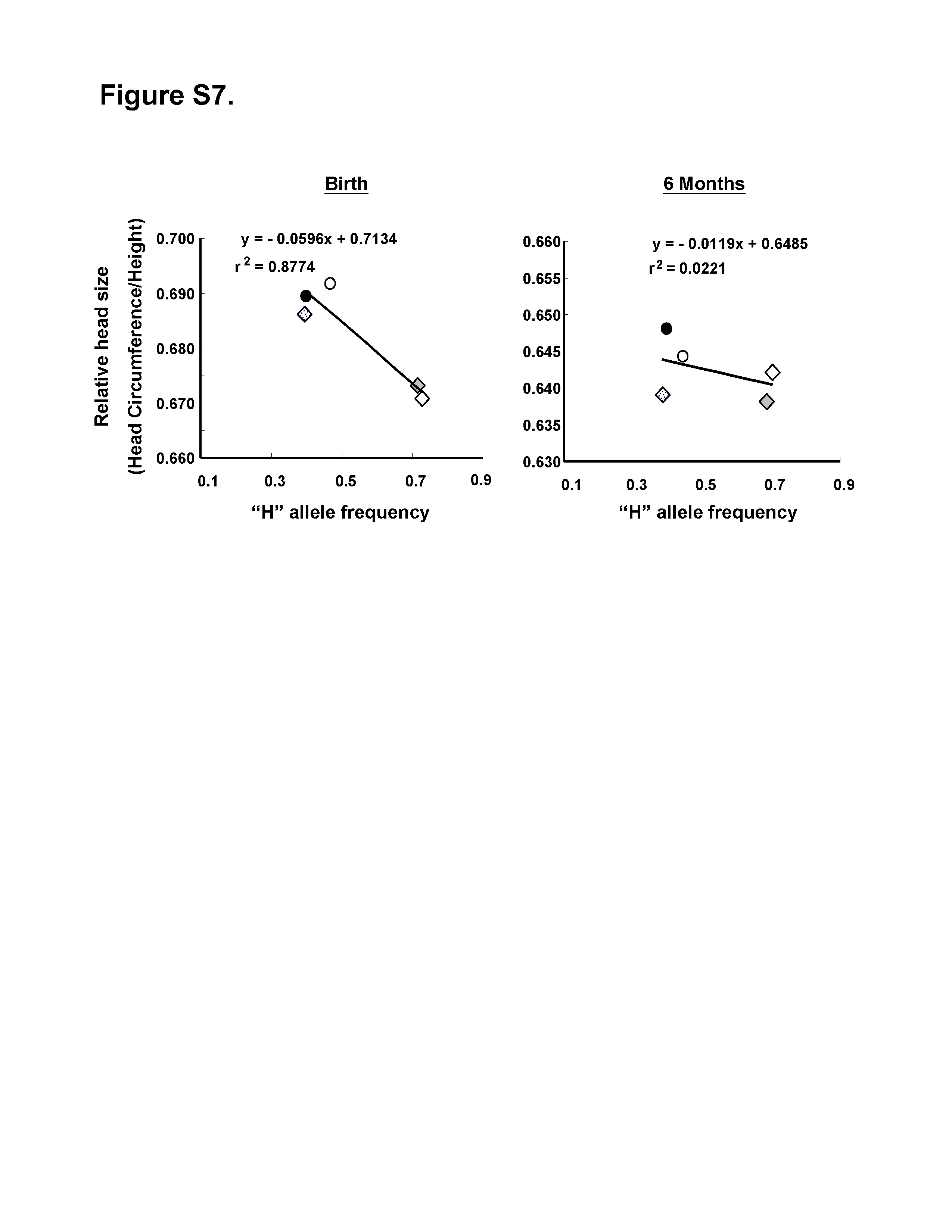

Supplement: Figure S7 — Associations between the H allele frequencies of M003-A06 and the relative head sizes of newborns among different ethnic groups. The relative head sizes of five ethnic groups were plotted against the frequencies of their H alleles. The H allele frequencies were extracted from the HapMap database. The head and height data were from the following sources: Japanese (open diamond), data from [35]; Chinese at Taiwan (closed diamond), data from Figure 6 ; Indians (closed circle), data from [64]; African Americans (stippled diamond), data from [65]; European Americans (open circle), data from [66]. Note the negative associations of the relative head sizes with the H allele frequencies at birth (p = 0.018, left panel), but not at the age of six months (p = 0.351, right panel). (TIF) [file pone.0047481.s007.tif]

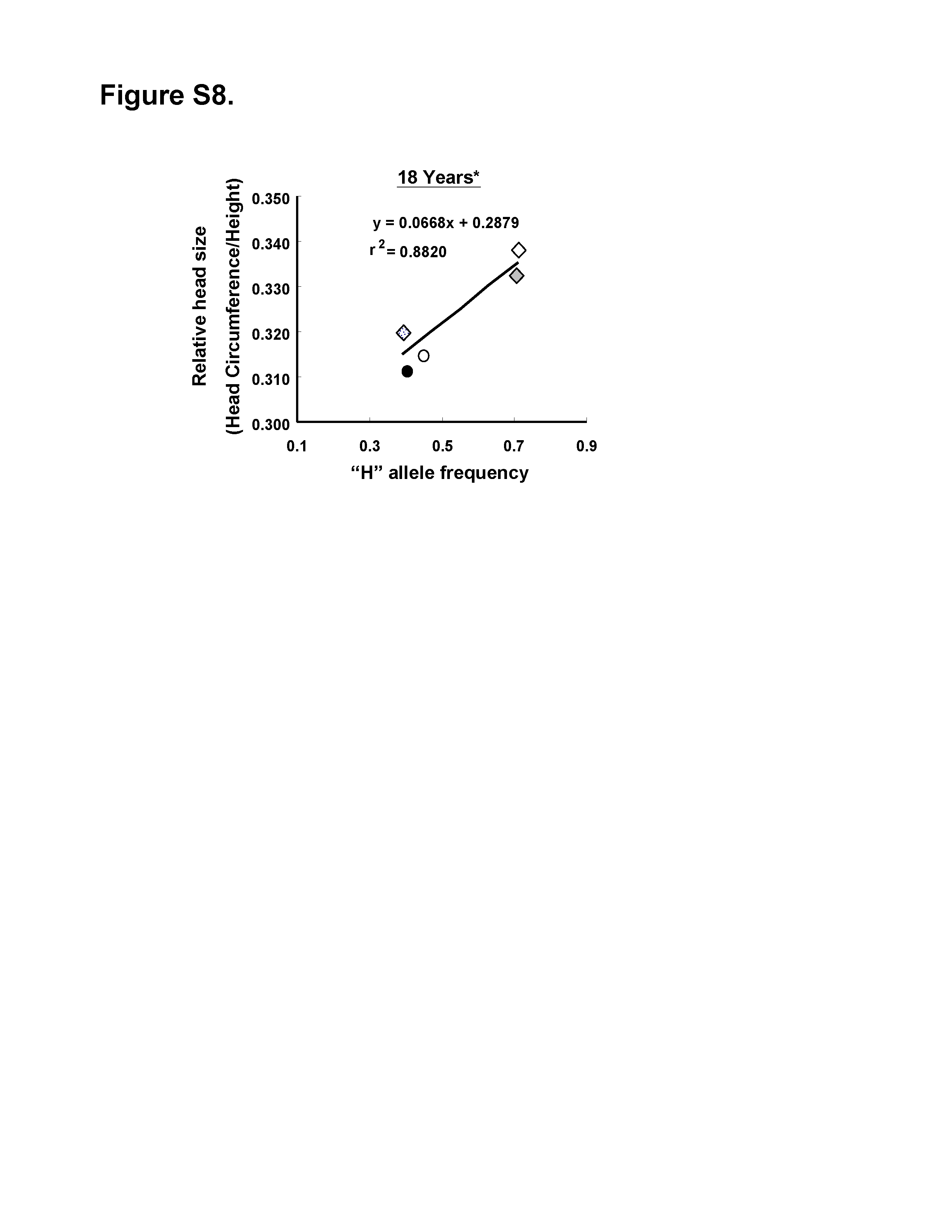

Supplement: Figure S8 — Associations between the H allele frequencies of M003-A06 and the relative head sizes of adult males among different ethnic groups. The relative head sizes of five ethnic groups are plotted against the frequencies of their H alleles. The H allele frequencies were extracted from the HapMap database. The head and height data were from the following sources: Japanese (open diamond), data from [35]; Chinese (closed diamond), data from http://www.hk-doctor.com/tool/html/TOC_E.htm; Indians (closed circle), data from [67]; African Americans (stippled diamond), data from [68]; European Americans (open circle), data from [35]. *, the data of 17-year old African Americans were used for the analysis. For the other 4 groups, those of the 18-year old males were used. Note the positive associations of the H allele frequencies with the relative head sizes of the 18-year old males (p = 0.018; this figure) as well as 18-year old females (data not shown). (TIF) [file pone.0047481.s008.tif]

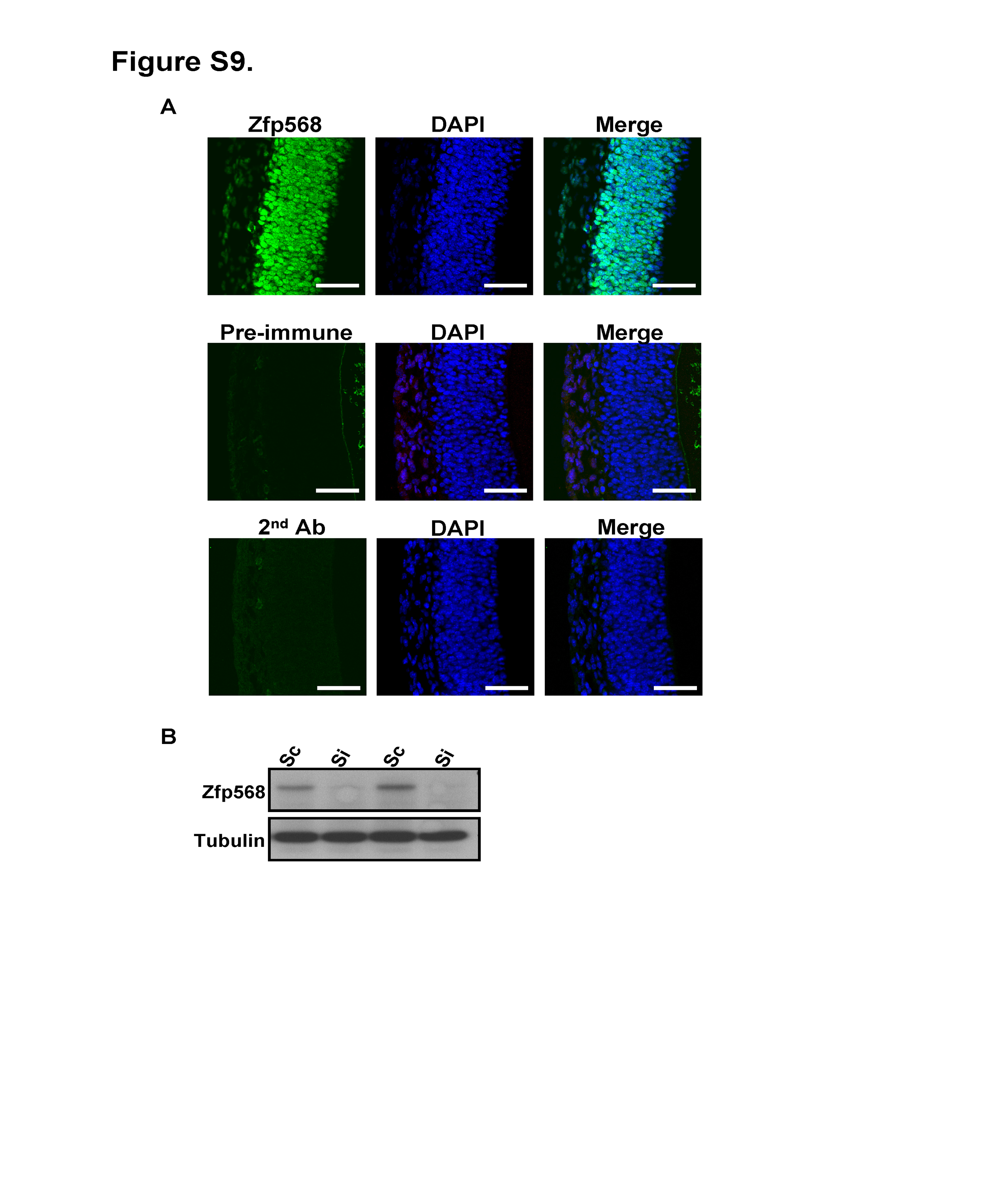

Supplement: Figure S9 — Specificity tests of the anti-Zfp568 antibody by immunofluorescence staining (A) and by Western blotting (B). (A) Co-staining patterns of the E12.5 brain sections with anti-Zfp568, the pre-immune rabbit serum (pre-immune), or the second antibody (Donkey anti-rabbit 488, 2nd Ab) with DAPI. Note the lack of signal from use of the pre-immune antibody (middle row) and the 2nd Ab (bottom row). Bars, 50 µm (B) Western blotting analysis, with use of anti-Zfp568, of extracts from Neuro2A cells transfected with either a scrambled control siRNA oligo (Sc) or a Zfp568-specific siRNA oligo (Si). Duplicated samples were used in the blottings. Tubulin was used as the loading control. (TIF) [file pone.0047481.s009.tif]
